# A computational framework for modeling and studying pertussis epidemiology and vaccination

**DOI:** 10.1186/s12859-020-03648-6

**Published:** 2020-09-16

**Authors:** Paolo Castagno, Simone Pernice, Gianni Ghetti, Massimiliano Povero, Lorenzo Pradelli, Daniela Paolotti, Gianfranco Balbo, Matteo Sereno, Marco Beccuti

**Affiliations:** 1 Department of Computer Science, University of Turin, Turin, Italy; 2 AdRes s.r.l, Turin, Italy; 3 Data Science for Social Impact and Sustainability, ISI Foundation, Turin, Italy

**Keywords:** Computational models, Colored Petri Nets, Epidemiological model, Pertussis

## Abstract

**Background:**

Emerging and re-emerging infectious diseases such as Zika, SARS, ncovid19 and Pertussis, pose a compelling challenge for epidemiologists due to their significant impact on global public health. In this context, computational models and computer simulations are one of the available research tools that epidemiologists can exploit to better understand the spreading characteristics of these diseases and to decide on vaccination policies, human interaction controls, and other social measures to counter, mitigate or simply delay the spread of the infectious diseases. Nevertheless, the construction of mathematical models for these diseases and their solutions remain a challenging tasks due to the fact that little effort has been devoted to the definition of a general framework easily accessible even by researchers without advanced modelling and mathematical skills.

**Results:**

In this paper we describe a new general modeling framework to study epidemiological systems, whose novelties and strengths are: (1) the use of a graphical formalism to simplify the model creation phase; (2) the implementation of an R package providing a friendly interface to access the analysis techniques implemented in the framework; (3) a high level of portability and reproducibility granted by the containerization of all analysis techniques implemented in the framework; (4) a well-defined schema and related infrastructure to allow users to easily integrate their own analysis workflow in the framework. Then, the effectiveness of this framework is showed through a case of study in which we investigate the pertussis epidemiology in Italy.

**Conclusions:**

We propose a new general modeling framework for the analysis of epidemiological systems, which exploits Petri Net graphical formalism, R environment, and Docker containerization to derive a tool easily accessible by any researcher even without advanced mathematical and computational skills. Moreover, the framework was implemented following the guidelines defined by Reproducible Bioinformatics Project so it guarantees reproducible analysis and makes simple the developed of new user-defined workflows.

## Background

Although in the last twenty years the human ability to efficiently treat infectious diseases has greatly improved, the latest pandemics of SARS and the Swine Flu outbreak have clearly highlighted how these diseases can spread faster in today’s interconnected world. In this context the computational epidemiology, a new multidisciplinary research field combining techniques from epidemiology, computer science, molecular biology and applied mathematics, makes extensive use of computational models for understanding and controlling spatio-temporal disease spread.

Roughly speaking, the computational models used in the study of infectious diseases at the population scale can be classified as *deterministic* and *stochastic*. In the first case, the system population is divided into small groups namely *compartments* (or classes) typically representing specific epidemic statuses [1–3]. These models are often formulated in terms of systems of differential equations (in continuous time) or difference equations (in discrete time), and produce an average description of the disease evolution at the population scale. Differently, stochastic models are formulated in terms of stochastic processes defined on families of random variables. These models capture in a straightforward manner demographic and environment variabilities and are useful in cases where randomness plays an important role. Typically they are formulated as Discrete Time Markov Chain (DTMC), Continuous Time Markov Chain (CTMC), and Systems of Systems of Stochastic Differential Equation (SDE) [4]. The choice between a deterministic model and a stochastic one depends on the application under study. For instance, deterministic models can be exploited to answer questions such as: *what fraction of individuals would be infected in an epidemic outbreak?, what conditions should be satisfied to prevent and control an epidemic?, what happens if individuals are mixed non-homogeneously?* [1], while the stochastic ones address questions such as: *how long is the disease likely to persist?, what is the probability of a major outbreak?* [4].

The construction of these types of models remains a challenging task. Indeed, despite of the large number of results published on this topic, little attention has been devoted to the definition of a general framework for modelling and studying infection diseases, which may be easily used by researchers without advanced computational skills. To the best of our knowledge, we believe that the only successful attempt to create a general framework for for modelling and studying infection diseases was proposed by Van den Broeck *et al* in [5]. Indeed all the other the works found in the literature, the analysis of systems combining population and disease characteristics, require the installation of many inter-dependent components to set up complex evaluation environments that are difficult to control and that make questionable the possibility of reproducing published results. Moreover, these workflows are often so specific that they can not be directly applied to analyze other models different from those for which they were originally developed.

To overcome these limitations and difficulties, we started the development of a general modelling and analysis framework with the objective of allowing researchers to better concentrate on the essence of these problems, and relieving them from the burden of setting up the complex environment needed for the solution of the complex mathematical models used for the investigation. Our modelling framework for studying epidemiological systems, shows novelties and strengths which can be summarized in: (1) the use of a graphical formalism based on Petri Nets [6*–*8] to simplify model construction and to provide an intuitive description of system behaviour; (2) the implementation of a R package to provide a user-friendly interface; (3) the containerization (into Docker images) of all the implemented analysis techniques to improve the framework portability and to ensure the reproducibility of the derived results; (4) the specification of a well-defined schema and related infrastructure to allow users to integrate their own analysis workflows in the framework.

The architecture of the framework reflects these features with the implementation of three modules that have been done taking into account the guidelines provided by the Reproducible Bioinformatics Project (RBP, http://reproducible-bioinformatics.org) a non-profit and open-source project, whose aim is to provide biologists and medical scientists with an easy-to-use and flexible environment for reproducible analysis.

The effectiveness of our proposal is shown with the investigation of Pertussis epidemiology in Italy. Specifically, we first point out that this framework can be easily used to develop an efficient workflow to analyse this very complex system.

Furthermore, we show that the model generated and calibrated according to such a workflow is able to reproduce real data coming from the observation of the spread of Pertussis in Italy during the period from 1974 to 2016. Moreover, we demonstrate that our framework can be easily exploited to support a what-if analysis on the model representing this complex system.

## Results

In this section, we first introduce the proposed framework in details, and then we show how it can be successfully used to study and analyze pertussis infection and the relative vaccination cycle in Italy.

### Modeling framework: a detailed overview

**Fig. 1:**
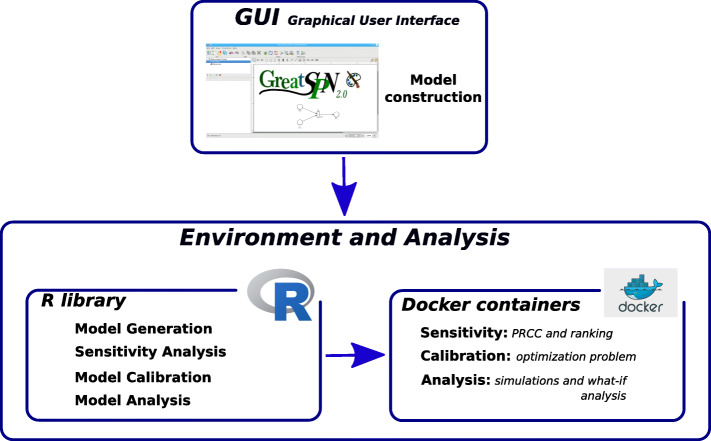
Framework schema depicting its modules and its functionalities from a user point of view

The architecture of this framework is composed of three main modules which cover different aspects of our proposal(see Fig. 1).

The first module consists of a Java Graphic User Interface (GUI) based on Java Swing Class which allows to draw models using the PN formalism. This graphical editor is part of GreatSPN [9], a software suite for modelling and analyzing complex systems using the PN formalism and its extensions. In particular, for the purposes of the framework presented in this paper, the GreatSPN GUI has been upgraded to support the Extended Stochastic Symmetric Net (ESSN), a high level Petri Net formalism, which enables users to define a system in a compact and parametric manner and to specify in a natural manner the rate functions which may be associated with the model reactions (The reader can find more details about the ESSN formalism in subsection *Petri Net and its generalization*).

The other two modules, consisting of an R library and a set of docker images, implement all the framework functionalities needed for the model analysis. Docker containerization, a *lightweight Operation System (OS)-level virtualization*, is exploited to simplify the distribution, the utilization and the maintenance of the analysis tools; the R library provides an easier user interface for which no knowledge on the docker commands is needed. Notice that all these docker images and R functions were created following the guidelines specified by RBP project to achieve a framework for developing reproducible workflow of analysis [10].

We now briefly describe all the functions implemented in the R library and their associated docker images.

The generation of the stochastic and deterministic processes underlying an ESSN model is implemented by the R function ***model_generation()***. This function automatically derives from the ESSN model the corresponding deterministic and stochastic processes using the C/C++ program *PN2ODE* embedded in the docker image *greatspn*. The derived processes and the library used to simulate them are packaged into a binary file with *.solver* extension. Currently the following solvers are available:

- *ODE solvers*: (1) Runge-Kutta 5th order integration, (2) Kutta-Merson integration; (3) Dormand and Princ method; (4) Backward Differentiation Formula (BDF) method;
- *Stochastic Simulation solvers*: (1) Gillespie algorithm; (2) Stochastic Hybrid simulation; (3) *τ*-leaping method.

More details on these solvers are reported in subsection *Implemented model solvers*.

The R function ***sensitivity_analysis()*** implements the sensitivity analysis starting from the *.solver* file generated by the ***model_generation*** function. This R function calls the R script *sensitivity.mngr.R* encapsulated into the docker image *epimod_sensitivity* to compute with the Partial Rank Correlation Coefficient (PRCC) analysis [11*,*^12^] the monotonic relationships between model inputs and outputs revealed (see “Monte Carlo sampling with PRCC” subsection for more details).

The model calibration is performed by the R function ***model_calibration()***. This function executes the R script *calibration.mngr.R* embedded in the docker image *epimod_calibration* that calls the right solvers according to the passed input parameter and produces as output a textual file in which all the generated parameter values are ranked according to their ability to fit the real data (i.e., from the best data fitting to the worst one). This is obtained solving an optimization problem in which the input objective function is minimized. More information on this aspect are reported in subsection *Implemented optimization solver to model calibration*.

Once the model is correctly calibrated, the R function ***model_analysis()*** solves the model and generates an output representing the time evolution of the model. The R script *model.mngr.R* embedded in the docker image *epimod_model* is then executed by ***model_analysis()*** function. Thus, this script simulates the underlying deterministic or stochastic process and returns a textual file in which the system solution is provided.

To ease the user in both experimentation and analysis of the model, our workflow encompasses a data visualization function. Specifically, the function ***display_data()*** offers a web application developed in Shiny providing a basic-level interface and an expert-level interface for data visualization. The basic-level interface consists of a simple but well-defined visualization environment, so that the user can directly focus on analyzing the results rather than spend its efforts setting up the necessary environment. Therefore, the web application enables the user to visualize the analysis results as line charts effectively while simplifying the process of generating plots to the extent that it is possible to visualize results with just few clicks. On the other hand, a simple visualization may not be enough to highlight complex behaviours of the system under study, and for this reason the function ***display_data()*** provides an additional expert-level interface which allows the user to implement its own visualization plots. In this case, the user is required to provide a function describing how the output data derived by analysis phase must be manipulated to be plotted. Hence, this functionality makes the data visualization very flexible and with loose restrictions –i.e., being compatible with *ggplot2* [13] R library and does not require any additional library.

The R function ***download_images()*** prepares the docker environment downloading the docker images needed by the framework.

### Framework installation

The installation of the workflow requires the downloading of the extended version of the GreatSPN editor at http://www.di.unito.it/~amparore/mc4cslta/editor.html, and the R library at https://github.com/qBioTurin/epimod.

### How to integrate a new function in the framework.

The customization of the framework is one of the strengths of this proposal since it provides the generalizations needed to use this same framework for other epidemiological studies different form that discussed in this paper. To this aim we describe in this subsection how new solution functionalities can be easily added in the framework. Practically, a user must firstly embed the new tool into a docker images following the tutorial reported at www.reproducible-bioinformatics.org/ in the section *“How to be part of the Reproducible Bioinformatics project”*. Secondly, he/she must provide an R function implementing an interface for the created docker images. To simplify the creation of such controlling function the R function *skeleton.R*, reported in the library, can be exploited as prototype. Then, any new R function and associated docker image must always be supported by an explanatory vignette, accessible online as html document, and by a set of test data accessible online as well. Finally, this new R function and associated docker image must be submitted to the info@reproducible-bioinformatics.org so that the RBP core team verifies the compliance of the new functionalities with the RBP guidelines. In our case, this protocol means that, once the framework has been certified by the RBP core team, every new addition or improvement must first be verified by the RBP organization before integrating it into the framework. More details on this task can be found in [10].

### The case study: an example of application of the framework

In this subsection we describe how the proposed framework can be exploited to study the Pertussis infection and its vaccination cycle in Italy. We first introduce the problem and then we show how a model of this complex system can be constructed.

#### The disease

Pertussis, also known as whooping cough, is a highly contagious infectious disease caused by the bacterium Bordetella Pertussis which colonizes the ciliated cells of the respiratory mucosa. It provokes an uncontrollable coughing which often makes breathing hard and which can possibly lead to serious complications including death. The first vaccine against Pertussis was developed already in the 1930s by pediatrician Leila Denmark. Despite this, Pertussis remains a challenging public health problem because many aspects of its infection, disease, and immunity are not completely understood yet.

Although the implementation of Pertussis vaccination programs in many countries has decreased substantially its diffusion and mortality, Pertussis has not been totally eliminated and Pertussis-related hospital admissions and fatalities are still evident, particularly in young infants [14].

Moreover, the European Centre for Disease Prevention and Control (ECDC) in its annual 2017 report [15] highlighted an increasing trend of Pertussis cases in EU, probably due to the decrease in vaccine effectiveness over time and pathogen adaptation [14*,*^16^*,*^17^].

#### State of the Art

In this context computational modelling can play an important role in providing insights on the drivers of Pertussis epidemiology, in investigating alternative explanations of the observed resurgence and in predicting potential effects of different vaccination strategies.

To these aims, several models were proposed in the literature since 1980s; for instance in [18*,*^19^], an age-structured model is exploited to analyse the possible effects of adopting different vaccination strategies in Australia. Other models expressed in terms of systems of differential equations are used to explain the duration of the Pertussis natural immunity [20], or the importance of age-structured contacts [21]. Differently in [22], a set of Partial Differential Equation (PDE)s, characterized by age and time dependent variables, is proposed to study the vaccination related changes that may have occurred for the pertussis epidemic in the Netherlands from 1996 to 1997. In [23] it is shown that a stochastic process can be used to better capture Pertussis vaccination behaviour, as well as the nature and degree of protection provided by the the acellular Pertussis vaccine (aP). Similarly, in [24*,*^25^] a stochastic process modelling Pertussis vaccination is presented for the analysis of the disease effect in different countries, respectively Massachusetts (United States) and Thailand. However, all of these works address only a subset of the specific peculiarities of the pertussis disease. In [26] the authors report the necessity of incorporating into a single model more details of the disease (e.g., the population age, the individual immunization level, …) to better match the real observed dynamics and to predict the outcome of vaccination measures [26].

#### A Model of Pertussis disease in Italy

The many aspects of the Pertussis disease and of the vaccination strategies can be conveniently represented by extending the classical Susceptible - Infectious - Recovered - Susceptible (Susceptible-Infected-Recovered-Susceptible (SIRS)) model. In particular, this new model considers a population in which each individual is described by her/his age (i.e., newborn, young, or adult), her/his level of immunization (i.e., resistance level), her/his vaccination status (i.e., how many doses were administered) and her/his health state (i.e., susceptible, infected, and recovered). The main system events are: the infection of a susceptible individual due to a contact with an infected one, the vaccination of an individual involving the administration of vaccine doses at different time points, and the recovering of an infected individual.

**Fig. 2:**
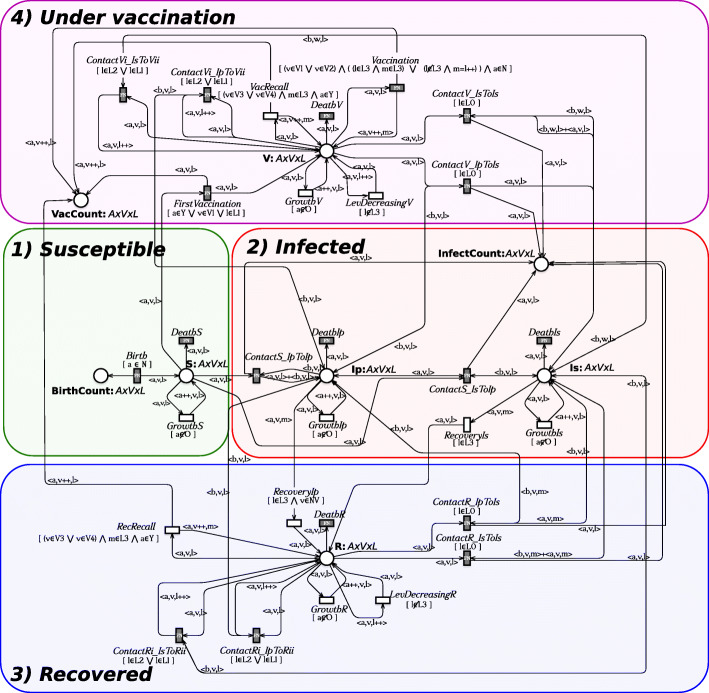
ESSN Model developed for studying Pertussis epidemiology and vaccination in Italy. It is divided in four sub-models representing the possible health states in which a person might be: *Susceptible*, *Under vaccination*, *Infected*, and *Recovered*

To keep under control the complexity of this phenomenon, the Extended Stochastic Symmetric Net (ESSN) formalism [6*,*^7^] is used. In Fig. 2 the ESSN model is showed. It consists of eight places and 30 transitions, and it is organized in four modules highlighted through colored boxes.

In details, places *BirthCount*,*VacCount*, and *InfectedCount* are introduced to count the total number of births, vaccinations, and infections happened during the system simulation. Hence, these places have a neutral domain and are introduced to make easier the computation of the measures of interest (e.g. the number of infected individuals in each year).

Places *S*, *V*, *Ip*, *Is*, and *R* encode the possible health states in which a population member may be (i.e., *Susceptible*, *Under vaccination*, *Infected due to primary infection*, *Infected due to repeated infection*, and *Recovered* respectively).

It is worth noting that the *Infected* state is modeled with two places to distinguish between individuals that are experiencing a primary infection (*Ip*) and those experiencing a repeated infection (*Is*). This distinction is important because primary and repeated infections have different characteristics [20].

The number of tokens in these places denotes the number of population members that are *Susceptible*, *Infected*, *Under vaccination*, and *Recovered* at any point in time, during the evolution of the system represented by the model. Moreover, each token in these places is labelled with the age, the level of immunization, and the vaccination status to better characterise each individual in the system. This is carried out defining the following three color classes:

- The class *A*={*a*_1_,*a*_2_,*a*_3_} records the age of a population member. It is divided in three static subclasses: *N*={*a*_1_} representing Newborn individuals (from 0∼11 months), *Y*={*a*_2_} representing Young individuals (11 months ∼18 years:), and *O*={*a*_3_} representing all the others (18∼99^+^ years).
- The class *V*={*v*_0_,*v*_1_,…,*v*_5_} represents how many vaccination doses were currently received. Since the Italian vaccination policy establishes three doses within the first 11 months of life followed by two additional boosters between 12 and 18 years of age, then we accordingly split this class in six static subclasses (i.e., *NV*={*v*_0_} no vaccination, *V*1={*v*_1_} first vaccination, … *V*5={*v*5} fifth vaccination).
- The class *L*={*l*_0_,…,*l*_3_} represents the ability of a individual to limit pathogen burden. It is divided into four static subclasses (i.e., *L*_0_={*l*_0_},…,*L*_3_={*l*_3_}) encoding an increasing level of resistance.

The color domain associated with these places is defined by the Cartesian product *A*×*V*×*L*. Moreover the transitions *GrowthS*, *GrowthIp*, *GrowthIs*, *GrowthR*, *GrowthV*, *RecRecall*, *RecoveryIp*, *LevDecreasingR* and *LevDecreasingV* are standard transitions (i.e following Mass Action (MA) law) while all the others are general transitions (i.e. whose rates are defined as general functions).

Observe that all the constants, the numerical values and the generic functions associated with these transitions are deeply described in the Additional file 1

The four modules corresponding to the four health states of an individual are now described.

**1) Supsceptible module.** It describes the behaviour of susceptible individuals. Transition *Birth* models the birth of a new person adding a new token in places *BirthCount* and *S*. Since a newborn enters into the system with the lowest level of resistance and without vaccination then the token added in place *S* is 〈*a*_1_,*v*_0_,*l*_0_〉. Differently, the age growth and the death of a susceptible individual are modeled by transitions *GrowthS* and *DeathS* respectively. Observe that the successor operator (i.e., *s*++) in the arc function labeling the output arc connecting *GrowthS* to *S* is used to represent the increasing of the age, while the guard [*a*∉*O*] associated with *GrowthS* guarantees that this transition is disabled when the maximum level of age (i.e., *O*) is reached.

**2) Infected module.** It models the behaviour of infected individuals. In particular, two types of infections, primary and repeated infections are considered and represented by places *Ip* and *Is*, respectively. Similarly to what done in the *Supsceptible* module, the age growth of an individual with primary (resp. repeated) infection is modeled by the transition *GrowthIp* (resp. *GrowthIs*), while the individual death is represented by the transition *DeathIp* (resp. *DeathIs*).

Transition *ContactS_IpToIp* (resp. *ContactS_IsToIp*) models the infection of a susceptible member due to a contact with one individual with primary (resp. repeated) infection. Thus its firing removes one token from S and adds it into *Ip*.

Finally, the recovery from a primary (resp. repeated) infection is modeled by transition *RecoveryIp* (resp. *RecoveryIs*), which removes one token from the place *I*_*p*_ (resp. *I*_*s*_) and adds it to the place *R*. In particular, the guards associated with these transitions (i.e., *RecoveryIp* and *RecoveryIs*) guarantee that the recovered patient has the highest level of immunity (i.e., [*l*∈*L*_3_]).

**3) Recovered module.** It describes the behaviour of recovered individuals. Transition *ContactRi_IpToRii* (resp. *ContactRi_IsToRii*) models the natural booster that increases to *l*_3_ the resistance level of a recovered with resistance level *l*_1_ or *l*_2_ after a contact with a individual with a primary (resp. repeated) infection.

These transitions (i.e. *ContactRi_IpToRii* and *ContactRi_IsToRii*) can fire only if *l* belongs to *L*_1_ or *L*_2_, guaranteed by the guard [*l*∈*L*_1_ || *l*∈*L*_2_]. Transition *ContactR_IpToIs* (resp. *ContactR_IsToIs*) describes the relapse of a recovered individual with the lowest resistance level (see guard [*l*∈*L*0]) due to the contact with a population member affected by a primary (resp. secondary) infection.

Transition *RecRecall* models the two vaccine recalls between 12 and 18 years old, which are possible only if all the previous three doses were successfully administrated during the first year of life. This is ensured by the guard [(*v*∈*V*_3_ || *v*∈*V*_4_) & *m*∈*L*_3_ & *a* ∈ *Y*], which enables the transition only if a individual is in the second age class (i.e *a* ∈ *Y*) with three (i.e *v*∈*V*_3_) or four (i.e *v*∈*V*_4_) vaccine doses already administrated. Thus, each administration increases the patient resistance level to its maximum (i.e. the transition guard *m*∈*L*_3_). Moreover, each time transition *RecRecall* fires, one token is added to the place *VacCount* for counting the number of vaccine doses whic have been administrated.

Transition *LevDecreasingR* represents the reduction of the resistance level. Observe that the immunization is totally lost after about 14 years [27] from the last infection. In particular, when the resistance level of an individual reaches the minimum value, i.e. [*l*∈*L*_0_], a recovered patient becomes again susceptible for infection. Her/his relapse is modeled by transitions *ContactR_IpToIs* and *ContactR_IsToIs* respectively. Finally, the age growth and the death of a recovered patient are encoded by transitions *GrowthR* and *DeathR*.

**4) Under vaccination module.** It implements the vaccination policy. Similarly to the recovered module, transitions *ContactV_IpToIs* and *ContactV_IsToIs* model the infection process, while transitions *ContactVi_IpToRii* and *ContactVi_IsToRii* the natural booster, *GrowthV* the aging and *DeathV* the death. Differently from the recovered module, the reduction of the resistance level obtained by the vaccine is lost after about 7 years [27]. This process is modeled by the *LevDecreasingV* transition. The starting of the vaccination process is represented by transition *FirstVaccination*, whose guard guarantees that vaccination is administrated only to a susceptible child. To complete the vaccination coverage, the administrations of two further doses are modeled by the *Vaccination* transition. Its guard, defined as [(*v*∈*V*_1_ || *v*∈*V*_2_) &((*l*∈*L*_3_ &*m*∈*L*_3_)||(*l*∉*L*_3_ &*m*=*l*++))& *a* ∈ *N*], guarantees that, under the condition to be in the first age class, (i.e. *a*∈*N*, only if the first or second vaccination is administrated) it is possible to move into the successive vaccination class, i.e. if *v*∈*V*_1_||*v*∈*V*_2_ then the output arc instance is characterized by *v*++. Indeed, the resistance level increases, due to the new dose administration, only if the level is not already at the maximum value, i.e. (*l*∈*L*_3_ &*m*∈*L*_3_) || (*l*∉*L*_3_ &*m*=*l*++). Finally, every time that transitions *FirstVaccination*, *Vaccination*, and *VaccRecall* fire, a new token is added to the place *VaccCount*.

#### A workflow for studying the Pertussis in Italy

**Fig. 3:**
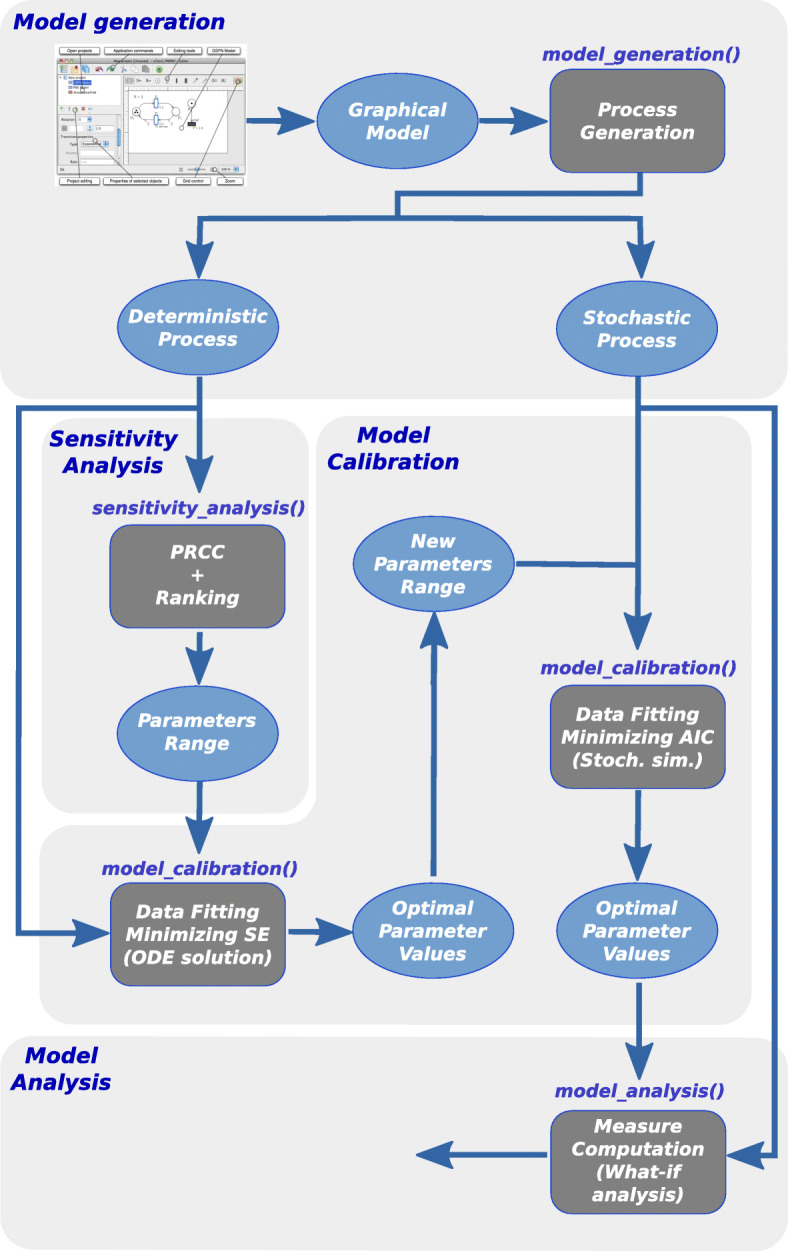
The schema of the workflow implemented for studying the ESSN model in Fig. 2

We now describe how the framework functions can be combined to obtain an analysis workflow for such model. This schema is summarized in Fig. 3 in which the light grey rectangles correspond to the four phases (i.e., *Model generation*, *Sensitivity Analysis*, *Model Calibration* and *Model Analysis*) implementing the analysis of our Pertussis model, while the dark grey boxes inside rectangles point out the main R framework functions exploited in each step of the analysis. The output of each task is instead highlighted by a blue circle.

**Model Generation.** The starting point of this workflow is the *Model Generation* phase, which derives from the Pertussis model the corresponding underlying stochastic and deterministic processes. This task can be achieved applying the R function *model_generation()* on the Pertussis ESSN model (see the Additional file 1 for more details on the used command line). Then the derived deterministic process is represented by a system of 179 Ordinary Differential Equation (ODE)s, while the derived stochastic process is characterized by 1965 possible events. The total execution time needed to derive the two processes and to create the *.solver* file requires less than one minute on Intel Core I7 2.60Ghz.

After this initial step, *Sensitivity Analysis* and *Model Calibration* are two pivotal steps to make our model consistent with real observed data.

**Sensitivity Analysis.** It allows to identify among the input parameters which are the sensitive ones (i.e., those that have a great effect on the model behaviour). This may simplify the calibration step reducing (1) the number of variables to be estimated and (2) the search space associated with each estimated parameter. In our case study, we identified 15 input parameters characterized by a high uncertainty due to their difficulty of being empirically measured. Specifically, three of them represent the probabilities of having (i) the *susceptible infection success*, i.e., the infection of a susceptible individual due to a contact with an infected individual, namely *prob_infectionS*, (ii) the *resistant infection success*, i.e., the infection of a vaccinated or recovered individual with the minimum resistance level due to a contact with an infected individual, namely *prob_infectionR_l1*, and finally (iii) *the natural boosts*, i.e., the restoring of the resistance level to the maximum when a person with resistance level different from the minimum level comes into contact with an infected individual, namely *prob_boost*. The others 12 parameters define the proportion of susceptible and recovered individuals for each pair of age class and resistance level in the initial marking. Given the partial information that we have on the spreading of the infection over the Italian population at the beginning of our study (estimated from ISTAT website [28] at the beginning of 1974 decreased by the average number of infected individuals during the same year) such proportion is used to define an initial detailed situation adequate for our modelling study and compatible with the available data^1^

Furthermore, to provide a measure of the sensitivity of these parameters the function *sensitivity_analysis()* was applied on the deterministic process previously generated and considering the period from 1974 to 1994, when the type of vaccine was the whole-cell Per-tussis (wP) vaccine. The choice of this time interval for this analysis allows us to simplify our model disabling the vaccination process, since the wP vaccine era is widely considered as a good surrogate for pre-vaccine era [20].

**Fig. 4:**
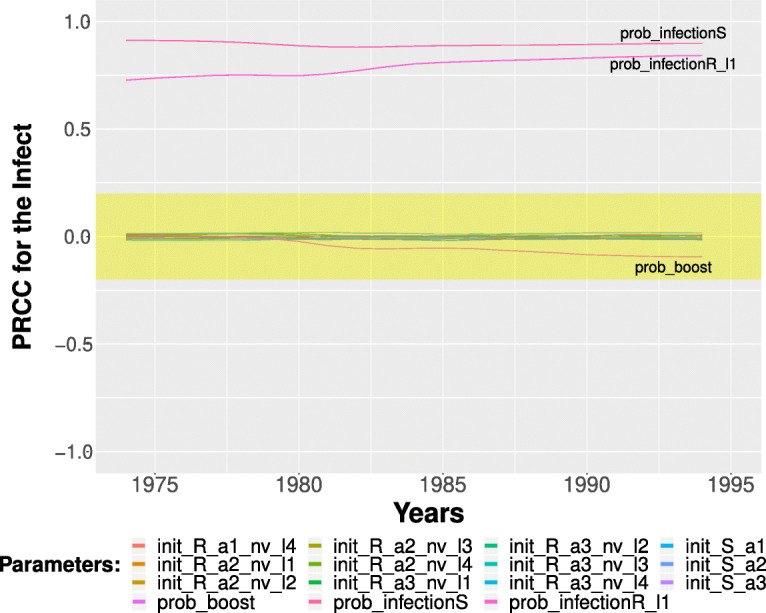
PRCCs values for the selected input parameters with respect the number of infections over the entire simulated period

Moreover, this model was run 64’000 times on this time interval: in every run a new input variable sample combination is generated according to the uniform distributions reported in Table 1, column two. Finally Partial Rank Correlation Coefficient (PRCC) between the generated input variables and the obtained model outputs (using Backward Differentiation Formula method for the numerical solution of ODE system) are evaluated. A complete description of the used command line is reported in the Additional file 1. The execution time for this analysis is ∼4h. on Intel Xeon processor @ 2GHz, exploiting a parallel execution on 40 cores. The computed results are reported in Fig. 4 in which the PRCCs values calculated for each parameter with respect to the number of infection cases over the entire time period are showed. From this plot it is straightforward to derive that the *prob_infectionS* is the most important parameter affecting the *infects* behaviour, followed by *prob_infectionR_l1*. Differently the *prob_boost* probability and the initial number of susceptible and recovered individuals in each age class are less relevant on the infection behaviour.

**Table 1:** Parameters variability range used during sensitivity and calibration analysis. In details, in the first column are listed the parameter names, then in the second and fourth columns the variability ranges used for the sensitivity and calibration analyses, respectively. The third column reports the initial parameters configuration. Finally, the fifth column is the optimal configuration discovered in the calibration analysis such that the quadratic error w.r.t. the real data is minimized

**Parameter name**	**PRCC ranges**	**GENSA Init.**	**GENSA ranges**	**GENSA Output**
prob_boost	[0,0.010]	0.0025	[0.0,0.0025]	0.002474758
prob_infectionS	[0,0.005]	0.0031	[0.0025,0.0100]	0.002537443
prob_infectionR_l1	[0,0.010]	0.0023	[0.0,0.0025]	0.002458887
init_S_a1	[0,866703]	866703	[0,866703]	866696
init_S_a2	[0,15685693	15685693	[0,15685693	15685680
init_S_a3	[0,37837299]	37837299	[0,37837299]	37628100
init_R_a1_nv_l4	[0,866703]	0	[0,866703]	7
init_R_a2_nv_l1	[0,15685693]	0	[0,15685693]	4
init_R_a2_nv_l2	[0,15685693]	0	[0,15685693]	2
init_R_a2_nv_l3	[0,15685693]	0	[0,15685693]	2
init_R_a2_nv_l4	[0,15685693]	0	[0,15685693]	2
init_R_a3_nv_l1	[0,37837299]	0	[0,37837299]	209184
init_R_a3_nv_l2	[0,37837299]	0	[0,37837299]	4
init_R_a3_nv_l3	[0,37837299]	0	[0,37837299]	4
init_R_a3_nv_l4	[0,37837299]	0	[0,37837299]	4

**Fig. 5:**
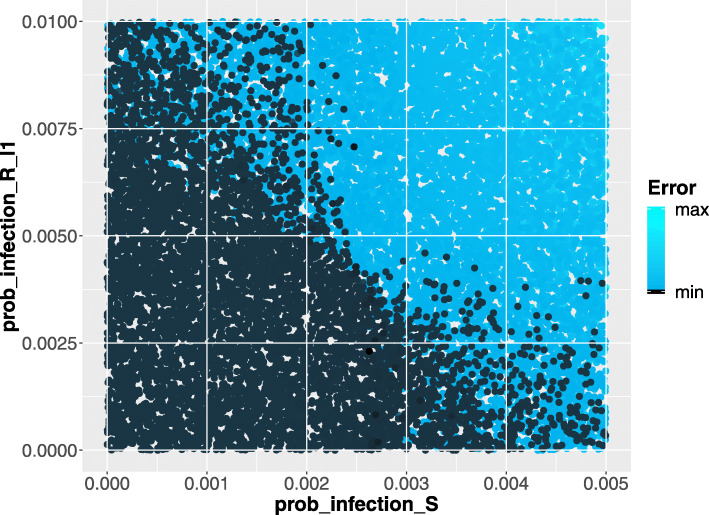
Scatter Plot showing the squared error between the real and simulated infection cases varying *prob_infectionS* and *prob_infectionR_l1*. The dark blue points represent the parameters configuration with minimum error w.r.t. the real data

In Fig. 5, the squared error between the real and simulated infection cases from 1974 to 1994 are plotted varying the *prob_infectionS* parameter (on the x-axis) and *prob_infectionR_l1* parameter (on the y-axis). Each point is then colored according to a linear gradient function starting from color dark blue (i.e., lower value) and moving to color light blue (i.e., higher values). From this plot we can observe that higher squared errors are obtained when *prob_infectionS* assumes values greater than 0.0025 and *prob_infection_l1* values greater than 0.005, see the light blue points within the region identified by values of *prob_infectionS* ∈[0.0025,0.005] and *prob_infection_l1* ∈[0.005,0.01]. Therefore, according to this we shrunk the search space associated with the two parameters in order to focus on the identified area.

**Model Calibration.** The aim of this phase is to adjust the model input parameters (e.g., *prob_infectionS*, *prob_infectionR_l1*, …) to have the best fit of simulated behaviours to the real data. As described in subsection *Modeling framework: a detailed overview*. our framework implements the calibration procedure through an optimization problem which minimises a user-defined object function. Since this optimization task is computationally expensive when a stochastic process is considered, we describe now a two-steps approach to speed-up this task that can be implemented easily using our R function. The idea behind this approach is to exploit the calibration of the deterministic process, typically faster, to reduce the parameter search space in the calibration of the stochastic process.

Then, in the first step the function *model_calibration()* is applied on the generated deterministic process to fit its behaviour to the real Italian infection data (from 1974 to 1994) using squared error estimator via trajectory matching, and then GenSA tool is executed to identify the best parameter set and Backward Differentiation Formula (BDF) method to solve the ODE system. Note that the information derived by the sensitivity analysis is exploited to reduce the number of parameters to be estimated and/or their search space.

**Fig. 6:**
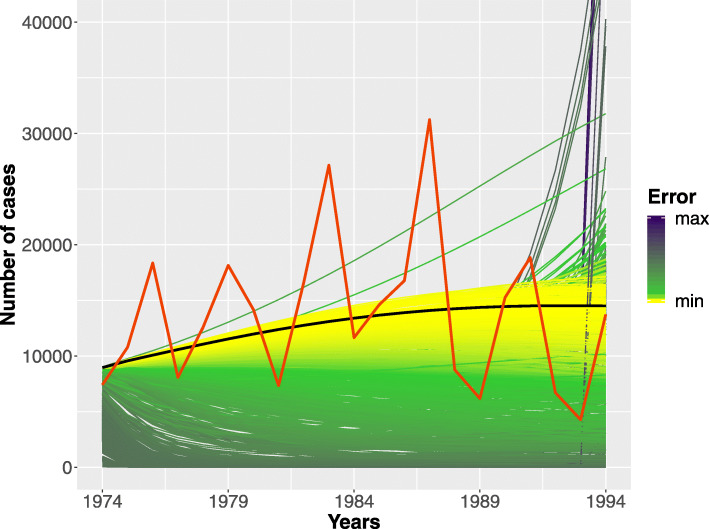
Model Calibration considering the deterministic model. Here a subset of the trajectories obtained from GenSA considering the parameter ranges stored in the fourth column of Table 1. The color of each trajectory depends on the squared error w.r.t. the Pertussis surveillance trend (red line). The black line is the optimal trend obtained by minimizing the squared error

Figure 6 shows a subset of all the trajectories generated by GenSA characterized by 15’000 trajectories extracted from a set of ∼ 90’000 trajectories obtained in ∼48h on an Intel Xeon processor @ 2GHz on a single core. The trajectories are colored depending on their distance (in terms of squared error) with respect to the Pertussis surveillance data (the red line). In details, the yellow color is associated with a low squared error, the purple color with a high squared error, while the optimal trajectory is showed in black. Moreover, the beam of trajectories (colored in yellow), closed to the optimal one, provides an indication on the ranges of parameter values that should be considered in the second steps of our calibration approach.

In the second step, the function ***model_calibration()*** is applied on the generated stochastic process to fit its behaviour to the real infection data using Akaike Information Criterion (Akaike Information Criterion (AIC)) via trajectory matching. The parameter search space of this second optimization step is then computed from the result obtained from the previous step, reported in the last column of the Table 1.

**Fig. 7:**
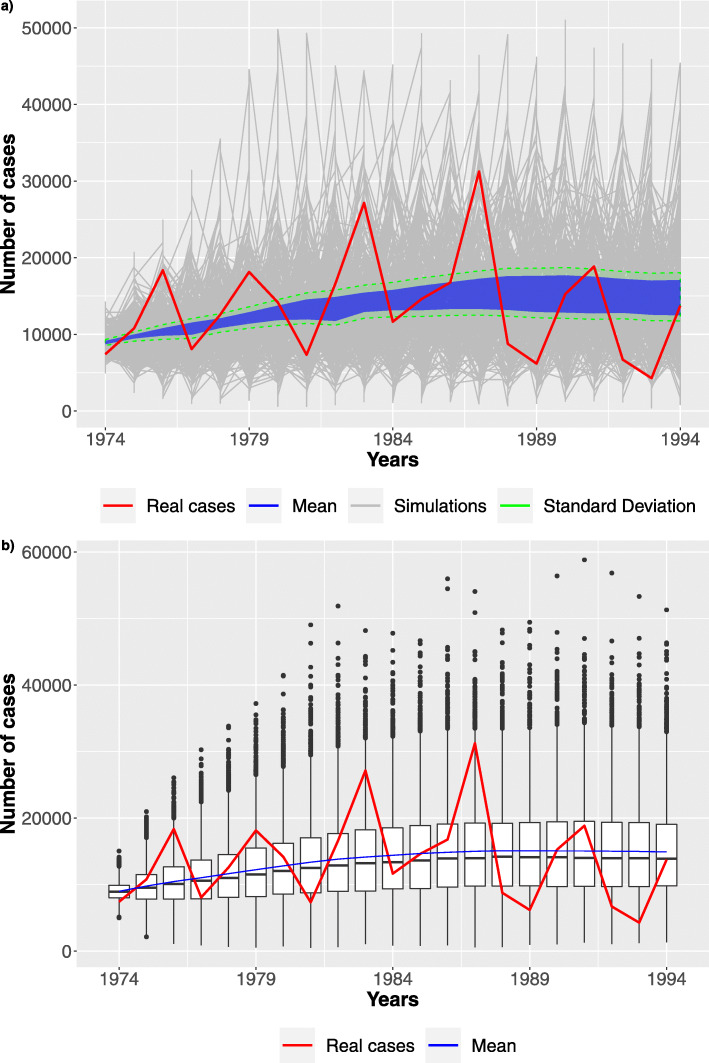
Stochastic simulations. **a** 4096 trajectories (grey) over the whole time interval are reported. **b** Boxplots over the time period considering the best configuration

Figure 7 shows trajectories (grey lines) for the fifteen best parameter configurations discovered, whose range values are reported in the Table 2. The blue area contains the average trajectories derived for the first ten best parameters configuration, while the two green lines provides the associated confidence interval. We can observe that a good approximation of the surveillance data (red line) from the 1974 to 1994 is obtained. This second step required about 48 hours on Intel Xeon processor @ 2GHz, exploiting a parallel execution on 40 cores. The trajectories are generated using the *τ*-leaping algorithm (see “Implemented model solvers” section for more details on this algorithm).

**Table 2:** Final parameters variability range used during the calibration of the model by solving the stochastic process *τ*-leaping algorithm

**Parameter name**	**Final range**
prob_boost	0.002523008∼0.002531240
prob_infectionS	0.002528196∼0.002529264
prob_infectionR_l1	0.002458931∼0.002474028
init_S_a1	866696
init_S_a2	15685680
init_S_a3	37628100
init_R_a1_nv_l4	7
init_R_a2_nv_l1	4
init_R_a2_nv_l2	2
init_R_a2_nv_l3	2
init_R_a2_nv_l4	2
init_R_a3_nv_l1	209184
init_R_a3_nv_l2	4
init_R_a3_nv_l3	4
init_R_a3_nv_l4	4

Finally, more details on the command lines used in these two phases are reported in the Additional file 1.

**Model Analysis** In this last phase of our workflow the user can analyse the calibrated model to answer specific questions and to derive new insights. In our case study we show a simple what-if analysis that can be implemented tacking advantage of the R function *model_analysis()*. In particular we investigate the impact of different vaccination failure probabilities with respect to the number of infection cases. The simulated time period is from 1974 to 2016, and the pertussis vaccination program is started in 1995, with an average vaccination coverage starts from 50% and transitions linearly to 95% in 8 years, [29*,*^30^]. The results are derived using the *τ*-leaping algorithm for generating 1024 trajectories for each case. The simulation of each case has required 4 hours on Intel Xeon processor @ 2GHz, exploiting a parallel execution on 40 cores.

**Fig. 8:**
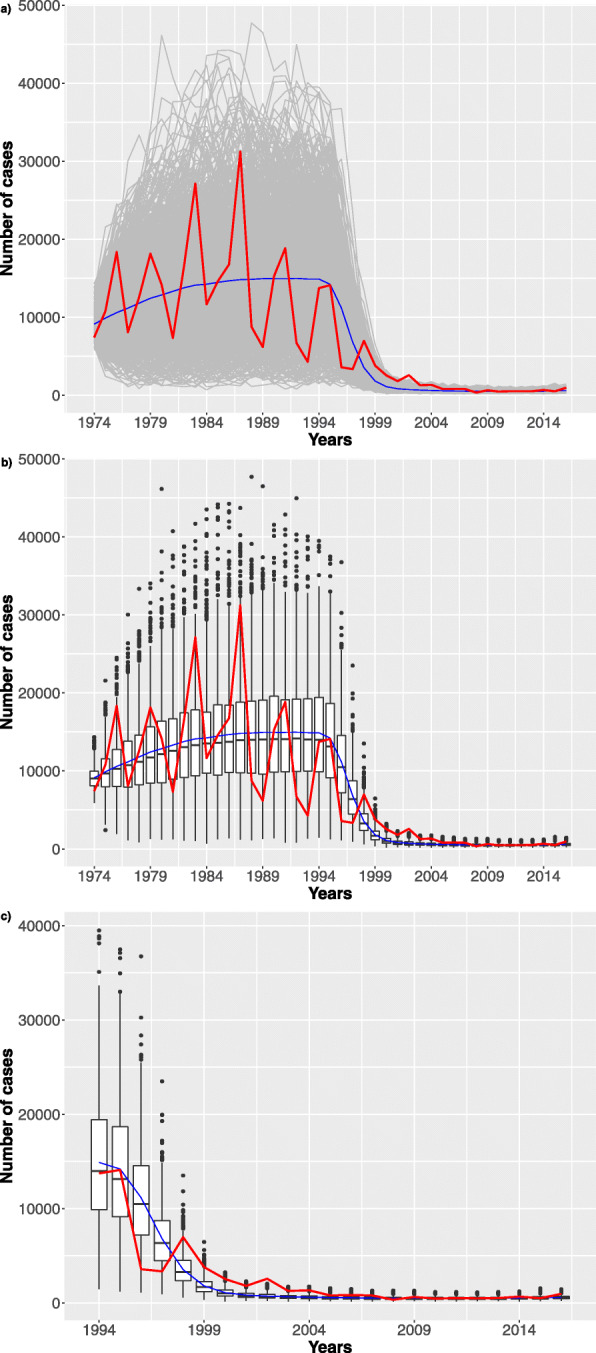
Stochastic simulations. Probability of vaccine failure settled to zero. **a** 1024 trajectories (grey) considering the stochastic model over the whole time interval. The blue dashed line represents the mean trend. Finally, the red line represents the Pertussis surveillance trend. **b** Boxplots over the time period. **c** Zoom considering the last 21 years

**Fig. 9:**
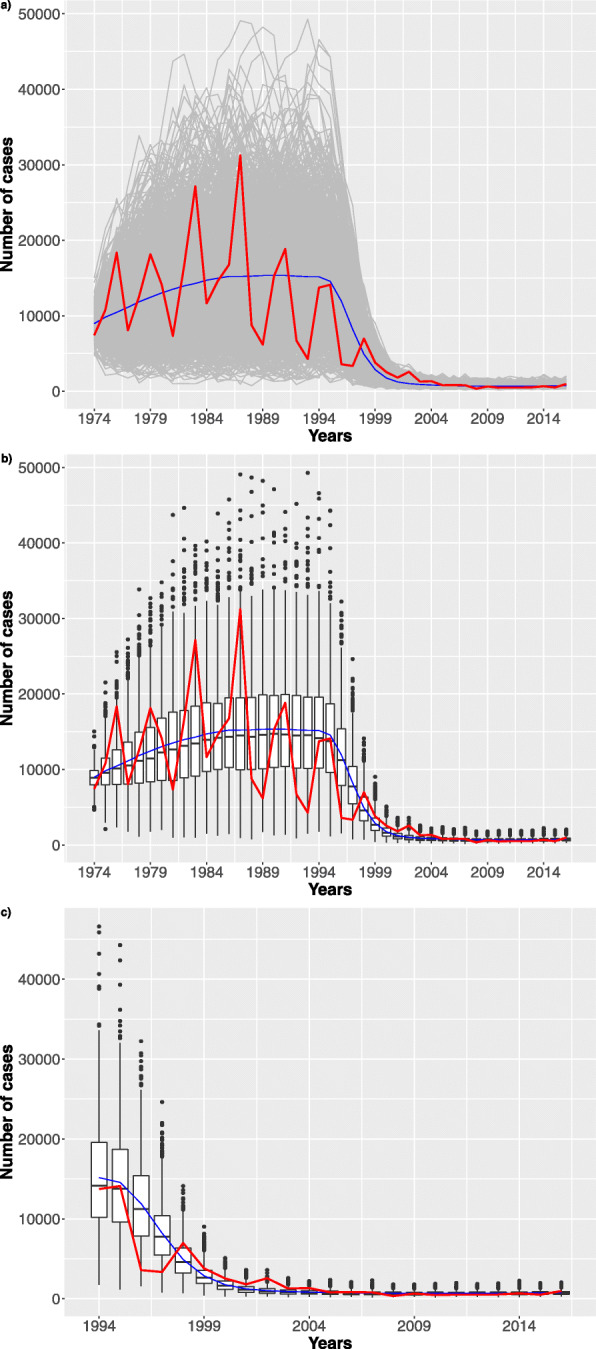
Stochastic simulations. Probability of vaccine failure settled to 10%. **a** 1024 trajectories (grey) considering the stochastic model over the whole time interval. The blue dashed line represents the mean trend. Finally, the red line represents the Pertussis surveillance trend. **b** Boxplots over the time period. **c** Zoom considering the last 21 years

**Fig. 10:**
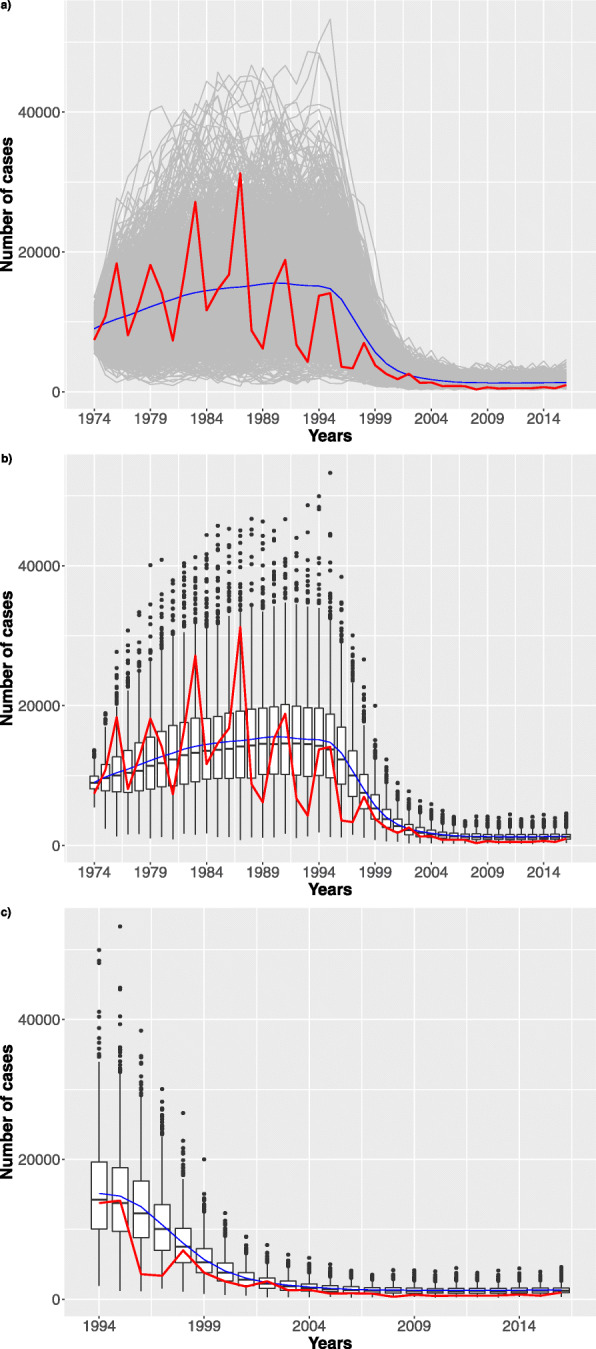
Stochastic simulations. Probability of vaccine failure settled to 40%. **a** 1024 trajectories (grey) considering the stochastic model over the whole time interval. The blue dashed line represents the mean trend. Finally, the red line represents the Pertussis surveillance trend. **b** Boxplots over the time period. **c** Zoom considering the last 21 years

In Figs. 8, 9, 10 we show how the number of infection cases is affected by increasing the vaccination failure probabilities from 0 to 0.5. We observed that only probabilities greater than 0.3 have an effect on the number of infection cases. For a matter of space, we only report results for failure probability of 0 (the reference), 0.1 and 0.4.

**Fig. 11:**
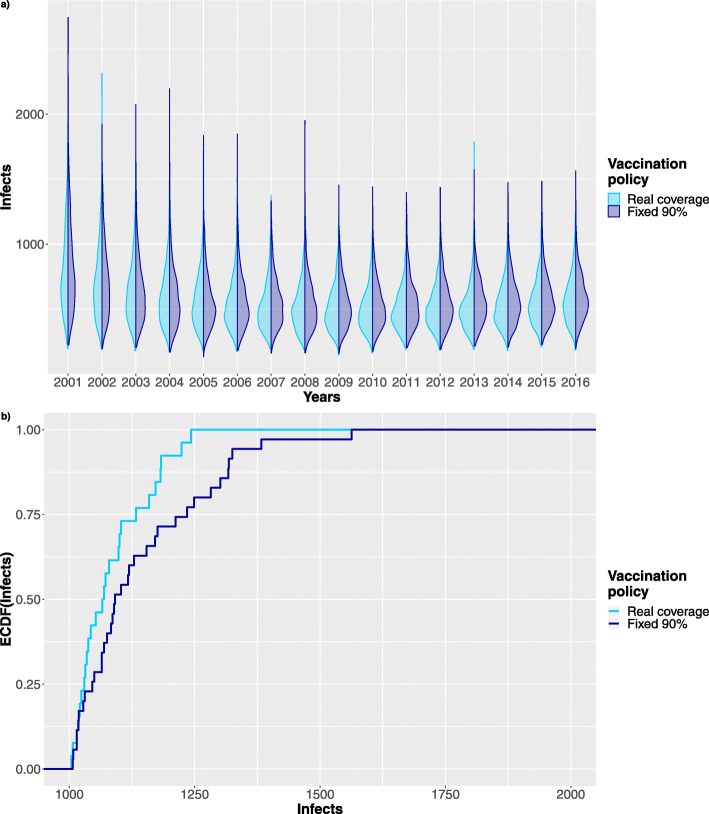
Stochastic simulations. Comparison between 1024 stochastic traces following the reference data and a scenario where the population vaccinated is reduced to the 90% starting from 2006. **a** Shows the violin plot comparing the distribution of infected patients in the two scenarios. **b** Comparing Infects ECDF after 10 years of reduced vaccination rate

**Fig. 12:**
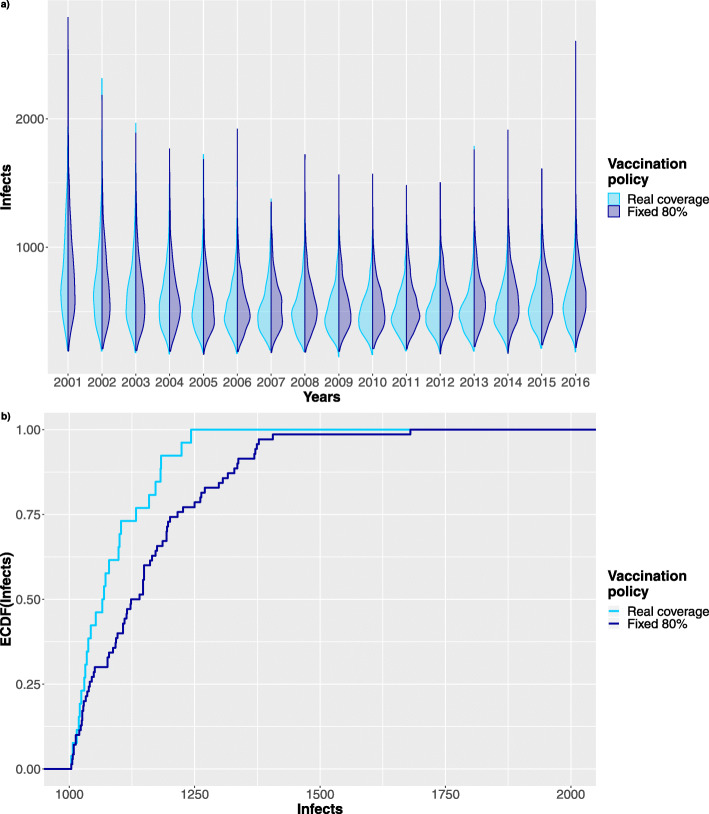
Stochastic simulations. Comparison between 1024 stochastic traces following the reference data and a scenario where the population vaccinated is reduced to the 80% starting from 2006. **a** Shows the violin plot comparing the distribution of infected patients in the two scenarios. **b** Comparing Infects ECDF after 10 years of reduced vaccination rate

Moreover, considering the same time period we further investigated the effects of varying the vaccination coverage of newborns in the period from 2006 to 2016. Figs. 11, 12 show results for vaccination coverage of 90% and 80% respectively. The simulation of each case comprises of 1024 stochastic traces and has required 4 hours on Intel Xeon processor @ 2GHz, exploiting a parallel execution on 40 cores.

In details Figs. 11a and 12a shows how the infects distribution shifts upward when the fraction of vaccinated newborns decreases.

Looking at the initial vaccination years (i.e. from 2001 to 2006) of these figures it is possible to notice that the distribution of infects look quite alike, as indeed they are the realizations of the same stochastic process. On the other end, starting from 2006 the two distributions begin to differ reflecting the changes in the vaccinated population.

Moreover, to better understand the effects on the distribution of infects in the population, Figs. 11b, 12b show the Empirical Cumulative Distribution Function (ECDF) of infects in 2016 for both the reference data series and the one with the percentage of vaccinated newborns reduced to 90% and to 80%. Comparing the two ECDFs it is clear that reducing the vaccination coverage the probability mass is shifted toward higher number of infects in the population. Indeed, the slope of the ECDF in Fig. 11b is much more steeper in the initial stage (i.e. in the range between 1000 and 1250) than that in Fig. 12b, meaning that a lower vaccination coverage remarkably increases the probability of having infection outbreak.

## Discussion

The health burden of well known infectious diseases was recently believed to become progressively negligible due to the fact that, among other factors, hygiene, improved nutrition, new drugs, and vaccination policies favoured a steady decline in overall mortality [31].

Quite the opposite, it is nowadays apparent that emerging and re-emerging infectious diseases such as Zika, Ebola, or Corona virus, pose a compelling challenge for epidemiologists; indeed human mortality attributed to infection is projected to remain at current levels of 13 to 15 million deaths annually until at least 2030, [31]. In this context, computational models and computer simulations are one of the available research tools that epidemiologists use to better understand the spreading characteristics of these diseases and to decide on vaccination policies, human interaction controls, and other social measures (including drastic) to counter, mitigate or simply delay the spread of the infectious disease. The construction of mathematical models of these diseases and their solutions remain however challenging tasks due to the fact that little effort has been devoted to the definition of a general framework easily accessible even by researchers without advanced modelling and mathematical skills. Despites of these needs and of the many studies reported in the literature to address these problems, to the best of our knowledge, we believe that the only successful attempt in this direction was GLEaM [5], a computational framework that exploits a stochastic model on a global population scale to simulate the large-scale spreading of influenza-like illnesses. Motivated by these considerations, we propose in this paper a new general modelling framework for the analysis of infectious diseases that does not require advanced mathematical computational skills for its utilization, and not even long and complex training phase for being used. The key issue underlying the development of our framework was to allow a domain expert (epidemiologist with limited knowledge of mathematical details) to use a simple, intuitive, but at the same time powerful tool to perform analysis and forecast on the spread of the disease, on the effect of vaccination campaigns, and/or on measures to contain the spread of the infection. Indeed, the use of a graphical formalism allows epidemiologists to conceive a model using a tool that is easier to handle than writing large sets of inter-related equations: Petri Net models are quite similar to the transmission flow diagrams widely used in epidemiology to describe the disease progressions. Then, the corresponding underlying deterministic and stochastic processes can be automatically generated and solved by our framework starting from the PN model. Indeed the framework provides a set of efficient and specific analysis techniques already integrated and ready-to-use. Differently a user should spend time to integrate existing solution methods or developed new ones. The novelties and strengths of the proposed framework with respect to GLEaM can be summarized as follows: (1) the use of a graphical formalism for the model creation; (2) a user-friendly interface based on R language; (3) framework portability and reproducibility of the results; (4) the possibility to integrate user-defined workflows. The effectiveness of this new framework was tested with a study of the pertussis epidemiology in Italy. The choice of this case study is due to the intrinsic complexity of the epidemiology and vaccination of this disease and to the need of comprehensive studies capable of addressing the many facets of this problem. Indeed, despite the fact that many models have been proposed since 1980s [18*–*23] with the aim of providing insights on vaccination strategies, duration of immunity, and epidemic episodes, all of them share the characteristics of addressing only a subset of the specific peculiarities of the pertussis disease, and none of them faces the necessity of incorporating into a single model more details of the disease (e.g., the population age, the individual immunization level, …) to better match the real observed dynamics and to predict the outcome of vaccination measures [26]. In subsection *A workflow for studying the Pertussis in Italy* we show that our framework can be easily exploited to construct and to analyse such a complex and comprehensive model (i.e., its underlying deterministic process is described by 179 ODEs and its underlying stochastic one is characterized by more than 1900 events). The development of such a model would be clearly unfeasible without the use of the graphical formalism; similarly, the analysis of such a representation would be difficult and error prone without the use of the suite of powerful solution tools integrated in the framework. As described in subsection *A workflow for studying the Pertussis in Italy*, the above model was calibrated in order to reproduce the observed Italian pertussis spread from 1974 to 2016. Figures 7a-b show that the model provides a good approximation of the real data giving confidence on the possibility of using it to answer specific biological questions such as the impact of different vaccine failure probability and/or different vaccination coverage on the probability to have a pertussis outbreak. This shows that focusing on the analysis of specific biological questions, a model of this type can be used to perform a what-if analysis to assess the sensitivity of the model to variations of certain input parameters. The high level of parametrization and the flexibility provided by the graphical formalism gives the possibility of re-using the model and its analysis workflows for many other cases beyond the one studied in this paper and represents one of the strengths of the proposed approach. With new contact matrices and new set of observed data, it would become possible to study other diseases or to model one disease with increasing levels of complexity/realistic ingredients. For instance we are adapting this model to investigate the effect of undetected infected individuals on the COVID-19 outbreak in Piedmont region. Although there are different patterns in the transmission and progression between the two diseases, there exist several building blocks in common between the two models and that helped us to develop, calibrate and analyze the new model in a matter of few weeks. For all these reasons we believe that this work can this proposed framework represents a substantial advance in the field of computational epidemiology and will be beneficial for the entire epidemiological community.

## Conclusion

In this paper we present a new general modeling framework for the analysis of epidemiological systems which exploits Petri Net graphical formalism, R environment, and Docker containerization to make easy its utilization even by researchers without advanced mathematical and computational skills. Moreover, the framework was implemented following the guidelines defined by Reproducible Bioinformatics Project, so that it provides reproducible analysis and makes simple the integration of new user-defined workflows. The effectiveness of this framework was then shown through a case of study in which we investigated the pertussis epidemiology in Italy.

## Methods

This section provides first a brief description of the sources of data utilized in our model, and of the Extended Stochastic Symmetric Net (ESSN) [6] formalism. Subsequently, we recall all the techniques implemented in our framework to perform the sensitivity analysis, the model calibration, and to evaluate the system behaviours.

### Data information

Pertussis notification data were collected from the Italian Ministry of Health [28*,*^32^] and Surveillance Atlas of Infectious Disease [33]. Such data report the number of Italian Pertussis cases per year from the beginning of 1974 until the end of 2016.

From the Italian Ministry of Health [34] we obtained the Italian population size, annual numbers of live births and deaths from 1974 to 2016. According to this we defined the birth and death rates as the average number of births and deaths, respectively, per day in each age class during the reference period.

The vaccine coverage data were extracted from [29] and [30]. Since the vaccine policy in Italy prescribes that three doses must be administrated within 11 months of age, the coverage at each year is defined as the proportion of children born that year who received three doses of the combined diphtheria, tetanus and aP vaccine (DTP) within 24 months of age.

The contact matrix depending on the three age ranges (*N*, *Y*and *O*) was estimated from that provided by [35], in which the Italian contact rates are reported assuming the population divided into 15 age ranges.

### Petri Net and its generalization

Petri Net (PN) [36] and their extensions are widely recognized to be a powerful tool for modeling and studying biological systems thanks to their ability of representing systems in a natural graphical manner and of allowing the computation of qualitative and quantitative information about the behavior of these systems.

In details, PNs are bipartite directed graphs with two types of nodes, namely *places* and *transitions*. The former ones correspond to state variables of the system and are graphically represented as circles. The latter ones correspond to the events that can generate a state change and are graphically represented as boxes. Nodes of different types are connected by *arcs*, which express the relation between states and event occurrences. A specific cardinality (multiplicity) is associated with each arc, and it describes the number of tokens removed from (or added to) the corresponding place upon the firing of the transition the arc is connected to. Graphically, it is written beside the arc, but the default value of one is omitted. Finally, places can contains *tokens* drawn as black dots. Then, the number of tokens in each place defines the state of a PN, called *marking*.

**Fig. 13:**
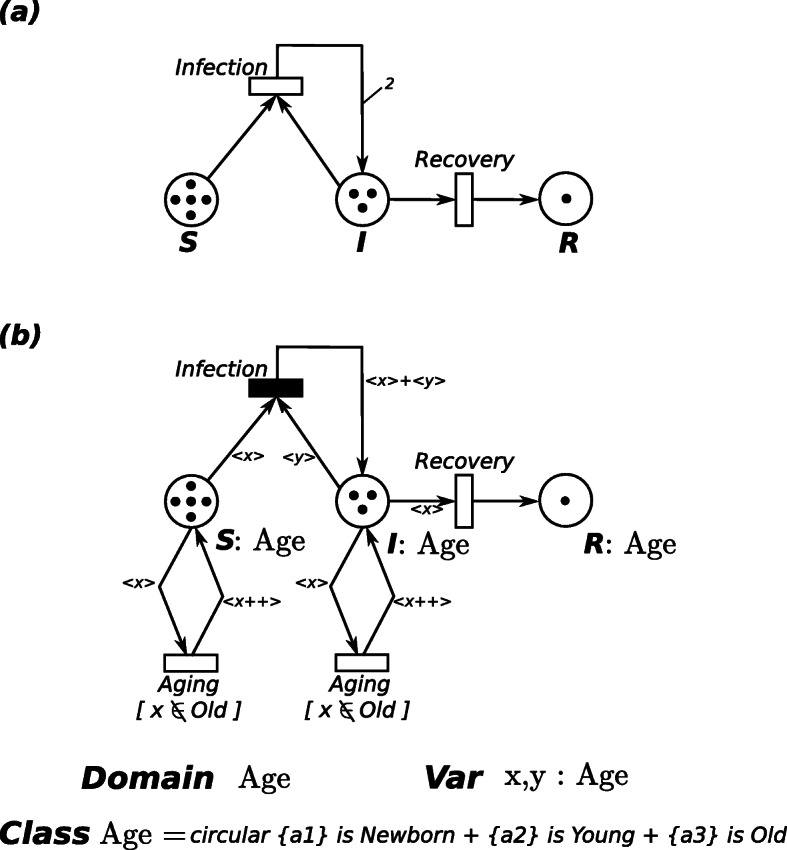
PN Examples of the simple SIR model. **a** PN formalism; **b** ESSN formalism

An example of a simple PN is given in Fig. 13a representing the classical Susceptible-Infected-Recovered (SIR) model. The places **S**, **I**, and **R** represent the three types of individuals that characterize the system, i.e. respectively susceptible, infected, and recovered. Then, the events that might occur are (i) the infection of a susceptible after the contact with an infected one, modeled by the transition *Infection*, and (ii) the recovery from the disease, represented by the transition *Recovery*. In Fig. 13a all the arcs have cardinality one, except the arc connecting the transition *Infection* to place **I** which has cardinality 2. The initial marking in Fig. 13a is defined as **S**〈5〉+**I**〈3〉+**R**〈1〉, meaning that the system is characterized by five susceptible individuals, three infected individuals and one recovered individual.

A transition is defined as *enabled* if and only if each input place contains a number of tokens greater or equal than a given threshold defined by the cardinality of the corresponding input arcs. Thus, the firing of an enabled transition removes a fixed number of tokens from its input places and adds a fixed number of tokens into its output places, according to the cardinality of its input/output arcs. In the Fig. 13a all the transitions are enabled in the initial marking. The system evolution is obtained from the firing of enabled transitions.

Among the PN generalisations proposed in literature, Stochastic Petri Net (SPN) represents a simple formalism that is relevant for the extensions used to specify the models considered in this paper. In SPNs delays are associated with transitions that, once enabled, take time to fire. Delays are specified as Negative Exponential random variables characterized by a rate parameter. The dynamic behavior of a SPN can be interpretded as a simple Stochastic Process which can be recognized as a CTMC.

ESSN [6*,*^7^] extends the SPN formalism allowing the users to easily define complex rate functions and providing a more compact, parametric, and readable representation of the system, due to the possibility of associating specific information (i.e. colors as in the Stochastic Symmetric Net (SSN) [8]) with each token. In the ESSNs, the set of transitions *T* is split in two sub-sets *T*_*ma*_ and *T*_*g*_, so that the former contains all transitions which fire with a rate following a MA law; the latter includes instead all the transitions whose random firing times have rates that are defined as general real functions. Transitions in *T*_*g*_ are graphically represented with black bar.

In details, each place *p* in the ESSN formalism has an associated color domain (i.e. a data type) denoted *cd*(*p*) and each token in a given place has a value defined by *cd*(*p*). Color domains are defined by the Cartesian product of elementary types called *color classes*, $\mathcal {C}=\left \{ C_{1}, \ldots, C_{n} \right \}$, which are finite and disjoint sets. They can be ordered (in this case a successor function ++ is defined on the class, inducing a circular order among the elements in the class), and can be partitioned into (static) subclasses.

For instance, the ESSN model in Fig. 13b extends the previous SIR model introducing the age of each population member through the color class *Age* divided into three subclasses *Newborn, Young*, and *Old*. Then, the color domain of all the places is *cd*(*S*)=*cd*(*I*)=*cd*(*R*)=*Age*

Each ESSN arc is labeled with an expression defined by the function *I*[*p*,*t*]: *cd*(*t*)→*Bag*[*cd*(*p*)], if the arc connects a place *p* to a transition *t*, while the opposite direction is defined by the function *O*[*p*,*t*]: *cd*(*t*)→*Bag*[*cd*(*p*)]. Where *Bag*[*A*] is the set of multisets built on set *A*, and if *b*∈*Bag*[*A*]∧*a*∈*A*, tyen *b*[*a*] denotes the multiplicity of *a* in the multiset *b*. In particular, the evaluation of *I*[*p*,*t*] (resp. *O*[*p*,*t*]), given a legal binding of *t*, provides the multiset of colored tokens that will be withdrawn from - input arc (resp. will be added to - output arc) the place connected to that arc by the firing of such transition instance.

Color domain are associated with transitions too. Considering a specific transition, its color domain is defined as a set of typed variables, where the variables are those appearing in the functions labeling the transition arcs and the variable types are the color classes. For instance, the color domain of transition *Infection* is *cd*(*Infection*)=*Age*×*Age* and the variables characterizing its input arc are *x*,*y*∈*Age*

An instance of a given transition *t* is an assignment of the transition variables to a specific color of a proper type. Hence, we use the notation 〈*t*,*c*〉 to denote an instance, where *c* is an assignment, also called *binding*. Moreover, a guard can be used to define restrictions on the allowed instances of a transition. A guard is a logical expression defined on the color domain of the transition, and its terms, called basic predicates, allow users (*i*) to compare colors assigned to variables of the same type (*x*=*y*, *x*≠*y*); (*ii*) to test whether a color element belongs to a given static subclass (*x*∈*C*_*i*,*j*_); (*iii*) to compare the static sub-classes of the colors assigned to two variables (*d*(*x*)=*d*(*y*),*d*(*x*)≠*d*(*y*)).

The *marking* of an ESSN is defined by the number of colored tokens in each place. For instance, a possible marking of the system of Fig. 13b can be: **S**(5〈*Newborn*〉)+**I**(4〈*Old*〉) representing a state with five supsceptible newborns and four infected old individuals.

Moreover, we denote with ^∙^**t** the set of input places of the transition *t* and with **t**^∙^ the set of output places of *t*, i.e. ^∙^**t**:={*p*∈*P*| ∃ *c*∈*cd*(*p*) *s*.*t*. *I*[*p*,**t**](*c*^′^)[*c*]>0} and **t**^∙^:={*p*∈*P*| ∃ *c*∈*cd*(*p*) *s*.*t*. *O*[*p*,**t**](*c*^′^)[*c*]>0}.

We use the notation *E*(*t*,*m*) to denote the set of all instances of *t* enabled in marking *m*. Where, in the case of the ESSN formalism, a transition instance 〈*t*,*c*〉 is enabled and can fire in an marking *m*, if: (1) its guard evaluated on *c* is true; (2) for each place *p* we have that *I*[*p*,*t*](*c*)≤*m*(*p*), where ≤ is the comparison operator among multisets. The firing of the enabled transition instance 〈*t*,*c*〉 in *m* produces a new marking *m*^′^ such that, for each place *p*, we have *m*^′^(*p*)=*m*(*p*)+*O*[*p*,*t*](*c*)−*I*[*p*,*t*](*c*).

In ESSNs each transition is associated with a specific rate, representing the parameter of the exponential distribution that characterises its firing time. Defining with $ \hat {m} (\nu)= m(\nu)_{|^{\bullet } \mathbf {t} }\ $ the subset of the marking *m*(*ν*) concerning only the input places to transition *t*, the parameter associated with an enabled transition instance 〈*t*,*c*〉 is given by the function

1 $$\begin{array}{*{20}l} F(\hat{m}(\nu),t,c,\nu) := & \left\{\begin{array}{ll} \varphi(\hat{m}(\nu),t,c), & t\in T_{ma},\\ f_{\langle{t,c}\rangle}(\hat{m}(\nu),\nu), & t\in T_{g}, \end{array}\right. \\  & \qquad \qquad \qquad f_{\langle{t,c}\rangle}\in\Omega(t,c) \end{array} $$

where *Ω*={*f*_〈*t*,*c*〉_}_*t*∈*T*∧*c*∈*cd*(*t*)_ is the set grouping all the real functions characterizing the transition speeds ∀*t*∈*T*, with *f*_〈*t*,*c*〉_=*φ*(*cdot*,*t*,*c*) when *t*∈*T*_*ma*_. Moreover *φ*(*m*(*ν*),*t*,*c*) is defined according to MA law as follows:

$$ \varphi(m(\nu),t,c)= \frac{\omega(t,c)}{I[p,t](c')[c]!} \prod_{\langle {p,c' }\rangle|\ p\in^{\bullet} \mathbf{t} \ \wedge\ c' \in \mathit{cd}(p)} m_{p_{j},c'}(\nu)^{I[p,t](c')[c]} $$

with *ω*(*t*,*c*) representing the rate of the enabled transition instance 〈*t*,*c*〉. Observe that $\varphi (\hat {m}(\nu),t,c)$ and $f_{\langle t,c \rangle }(\hat {m}(\nu),\nu)$ can depend only on the time *ν* and the marking of the input places of transition *t* at time *ν*.

As for the SPNs, also in the ESSNs the stochastic firing delays, sampled from negative exponential distributions, allow to automatically derive the underlying CTMC that can be studied to quantitatively evaluate the system behaviour [36]. In details, the CTMC state space, $\mathbb {S}$, corresponds to the reachability set of the corresponding ESSN, i.e. all the possible markings that can be reached from the initial marking. Thus, the Chapman-Kolmogorov equations (also called Master Equation) for the CTMC are defined as follow:

2 $$ \frac{d\pi(m_{i},\nu)}{d\nu} = \sum\limits_{m_{k}} \pi(m_{k},\nu)q_{m_{k},m_{i}} \qquad m_{i},m_{k} \in \mathbb{S}  $$

where *π*(*m*_*i*_,*ν*) represents the probability to be in marking *m*_*i*_ at time *ν*, and $q_{m_{k},m_{i}}$ the velocity to reach the marking *m*_*i*_ from *m*_*k*_, defined as

$$q_{m_{k},m_{i}}= \sum\limits_{ \substack{\mathbf{t} \in T \wedge \\ T\langle{\mathbf{t},\mathbf{c}'\rangle} \in E(\mathbf{t},m_{k})_{|m_{i}}} } F(m_{k},\mathbf{t},\mathbf{c}',\nu)\left(L[p,\mathbf{t}](\mathbf{c}')[c]\right). $$

where $E\left (\mathbf {t},m_{k}\right)_{|m_{i}}$ is the set of all instances of **t** enabled in marking *m*_*k*_ whose firing brings to the marking *m*_*k*_, and *L*[*p*,**t**](**c**^′^)[*c*]=*O*[*p*,**t**](**c**^′^)[*c*]−*I*[*p*,**t**](**c**^′^)[*c*].

In complex systems, the System of differential equations represented by the Master Equation (2) is often mathematically intractable (i.e it requires an equation for each system state), thus Monte Carlo simulation can be exploited to study the system behaviour. Let us underline that each trajectory obtained by Monte Carlo simulation represents one sample of the probability mass function that solves the Master Equation.

In case of very complex models, when the system stochasticity is negligible, then it is possible to exploit the so-called deterministic approach [37] which approximates the system behaviours through a deterministic process. This deterministic process is then described through a system of ODEs having one equation for each possible colored tuple *c* in each place domain (i.e. ∀*p*∈*P*,∀*c*∈*cd*(*p*)). Let us highlight that the deterministic process derived in this manner is able to well approximate the stochastic behavior of an ESSN model, if the CTMC underlying the model is a density dependent process, i.e., if all the transition rates belonging to *Ω* are represented by density dependent functions (see [38] for more details)

Let $x_{p,c}(\nu) \in \mathbb {R}^{+}$ be the continuous approximation of the number of tokens in place *p* and colors *c* so that the vector $x(\nu) \in \mathbb {R}^{n}\ $, is the continuous approximation of an ESSN marking at time *ν*.

Let also define $ \hat {x} (\nu)= x(\nu)_{|^{\bullet } \mathbf {t} }\ $ as the subset of the marking *x*(*ν*) concerning only the input places to the transition *t*, then the Eq. (1) becomes

3 $$\begin{array}{*{20}l} F(\hat{x}(\nu),t,c,\nu) := & \left\{ \begin{array}{llll} \varphi(\hat{x}(\nu),t,c), & t\in T_{ma},\\ f_{\langle{t,c}\rangle}(\hat{x}(\nu),\nu), & t\in T_{g}, \end{array}\right. \\  & \qquad \qquad \qquad f_{\langle{t,c}\rangle}\in\Omega(t,c). \end{array} $$

Finally the ODE characterizing the *p* and color tuple *c*∈*cd*(*p*) is defined as:

4 $$\begin{array}{*{20}l} \frac{dx_{p,c}(\nu)}{d\nu} &=  \sum\limits_{ \substack{\mathbf{t} \in T \wedge \\ T\langle{\mathbf{t},\mathbf{c}'}\rangle \in E\left(\mathbf{t},m_{k}\right)_{|m_{i}}} }  F\left(\hat{x}(\nu),\mathbf{t},\mathbf{c}',\nu \right)\left(L[p,\mathbf{t}](\mathbf{c}')[c]\right)  \\ &=  \sum\limits_{\substack{ \mathbf{t} \in T_{ma}\, \wedge \\ \langle{\mathbf{t},\mathbf{c}'}\rangle \in E\left(\mathbf{t},x(\nu)\right) }}  \varphi\left(\hat{x}(\nu),\mathbf{t},\mathbf{c}'\right)\left(L[p,\mathbf{t}](\mathbf{c}')[c]\right) \\ & +  \sum\limits_{\substack{ \mathbf{t} \in T_{g} \wedge \\ \langle{\mathbf{t},\mathbf{c}'}\rangle \in E(\mathbf{t},x(\nu)) }}  f_{\langle{\mathbf{t},\mathbf{c}'}\rangle}\left(\hat{x}(\nu),\nu \right)\left(L[p,\mathbf{t}](\mathbf{c}')[c]\right) \end{array} $$

where $\hat {x}(\nu)=x(\nu)_{|^{\bullet } \mathbf {t} }$.

### Monte Carlo sampling with PRCC

Sensitivity analysis is a well-known approach exploited in computational modeling to investigate which parameters affect mostly the variability of the outcomes generated by the model. In the literature several approaches are proposed to achieve this task, such as Pearson correlation coefficient (CC) method (for linear relationships), Partial Rank Correlation Coefficient (PRCC) method (for non-linear and monotonic relationships), and Fourier Amplitude Sensitivity Test (FAST) method (for any non-linear relationships) [11*,*^12^]. In this framework we implemented a sampling-based method which combines Monte CarloSampling MCS with PRCC index.

In details MCS is exploited to generate the samples of the model input variables. Then the model is run *N* times on a fixed temporal interval: one for each generated input variable sample combination. Finally, PRCC between the generated input variables and the obtained model outputs are evaluated on the same chosen interval. In this way the PRCC analysis and corresponding significance tests (i.e significant *p*-value) are utilized to identify key model parameters and to select time points which need an additional in-depth investigation. Specifically, PRCC values close to 1 (resp. -1) identify positive (resp. negative) monotone relationships between inputs and outputs; while the significance tests allow to discover those correlations that are important, despite having relatively small PRCC values.

### Implemented model solvers

In the literature many algorithms are proposed for the numerical solution of ODEs systems and for numerically generating time trajectories of a stochastic process. Obviously, each method has its strengths and weaknesses, and for these reasons we decided to integrate more than one algorithm in our framework. In detail, for the numerical solution of ODEs systems we implemented three explicit methods (i.e., *Runge-Kutta 5*^*th*^ order integration, *Dormand-Prince method*, and *Kutta-Merson method*) which can be efficiently used for systems without stiffness (i.e., the system solution is numerically stable) [39]. Instead for systems with stiffness we provided a Backward Differentiation Formula (Backward Differentiation Formula BDF) method [39] that we implemented using the C++ LSODA library (https://en.smath.com/view/lsoda)

For the simulation of the stochastic process, we implemented the Gillespie algorithm, called *Stochastic Simulation Algorithm* Stochastic Simulation AlgorithmSSA [40], the *τ*-leaping method [41] and *Stochastic Hybrid simulation* Stochastic Hybrid simulation SHS. The SSA is an exact stochastic method widely used to simulate chemical systems whose behaviour can be described by the Master Equations, Eq. 2. In case of very large systems (i.e., systems with a large numbers of interacting elements) SSA could be computationally too slow, and then approximation methods must be used. Among these approaches the $\mathbb {\tau }$*-leaping algorithm* provides a good compromise between the solution execution time and its quality. Indeed, this method speeds up the stochastic simulation of system by approximating the number of system events during a chosen time increment (i.e., *τ*) as a Poisson random variable. Another approximation method implemented in our framework is the *Stochastic Hybrid Simulation* (SHS), based on the co-simulation of discrete and continuous events [42]. This approach provides a speed-up under the assumption that all the faster events are modeled as continuous. Currently the user has to statically provide the splitting between discrete and continuous events associating with them a specific label that can be represented in the model using the GreatSPN GUI.

### Implemented optimization solver to model calibration

In Computer Science, Mathematics, and Operations Research, optimization or mathematical programming consists of minimizing (or maximizing) a function by consistently selecting the values of its variables from a set of feasible possibilities utilizing analytical or numerical methods. Formally an Optimization Problem (OP) with inequality constrains can be defined as follows:

$$\begin{aligned} & \underset{\mathbf{x}}{\text{minimize}} & & \mathcal{F}_{opt}(\mathbf{x})\\ & \text{subject to} & & \mathcal{G}_{i}(\mathbf{x}) \geq b_{i}, \;\;\;\; 1\leq i \leq l\\ & & & \mathcal{L}_{i}(\mathbf{x}) \leq c_{j},\;\;\; 1\leq j \leq m\\ \end{aligned} $$

where the vector **x**=(*y*_1_,…,*y*_*n*_) is the *variable vector*, the function $\mathcal {F}_{opt}: \mathbb {R}^{n} \rightarrow \mathbb {R}$ is the *objective function*, the functions $\mathcal {G}_{i}(\mathbf {x}): \mathbb {R}^{n} \rightarrow \mathbb {R}$ and $ \mathcal {L}_{i}(\mathbf {x}): \mathbb {R}^{n} \rightarrow \mathbb {R}$ are *inequality constraint functions*, and the constants $b_{1},\dots,b_{l},c_{1},\dots,c_{m}$ are the *bounds* for the constraints. A vector **x**^∙^, called *optimal*, is the solution of the OP if, among all vectors that satisfy the constraints, it is that which yields the smallest (largest) value of the optimization function: ∀**z** s.t. $\mathcal {G}_{1}(\mathbf {z}) \geq b_{1},\ldots, \mathcal {L}_{1}(\mathbf {z}) \leq c_{m}$ we have that $\mathcal {F}_{opt}(\mathbf {z})\geq \mathcal {F}_{opt}(\mathbf {x}^{\bullet })$.

OP is termed a *linear program* if the objective and constraint functions are linear and *non-linear* otherwise. In our framework, the focus is on non-linear programs in which constraints can be non-linear as well. To solve this type of OPs, several algorithms have been proposed in the literature, an overview on these methods is reported in [43]. Among the available algorithms, the one integrated in our framework is the Generalized Simulated Annealing for Global Optimization implemented in the R package GenSA [44], since it was designed to solve complicated nonlinear objective functions with a large number of local minima. Moreover, we are currently evaluating the integration of new optimization algorithm based on deep learning and Neural network.

### Docker containerisation in a nutshell

Container technology, a lightweight Operation System (OS)-level virtualization, was recently proposed in the area of Bioinformatics as an efficient solution to simplify the distribution, the usage and the maintenance of bioinformatics software [45]. Indeed, the users exploiting containerization have not to deal with dependency or compilation problems; since an applications and their dependencies are already packaged and installed together into the container image. Obviously, this simplifies considerably the installation and the usage of the applications encapsulated into a container image. Among the container platforms proposed in literature, Docker (http://www.docker.com) is getting actually the standard environment to quickly build, deploy, scale and manage containerized applications under Linux. In summary docker strengths are its high level of portability, which allows users to easily register and share containers over different hosts, and to achieve a more effective resource use and a faster deployment compared with other similar software.

## Supplementary information

**Additional file 1** A pdf file providing more information on the usage of the proposed framework, in particular the whole Pertussis analysis is showed. Furthermore, a simpler example of analysis is exploited as a step by step guide of the EPIMOD package.

## Data Availability

All data generated and analyzed during this study are included in this published article and its Additional file 1. Moreover, all the R files and the GreatSPN file of the net are freely available at https://github.com/qBioTurin/epimod/.

## Abbreviations

CTMC: Continuous time Markov chain
DTMC: Discrete time Markov chain
MA: Mass action
PN: Petri net
SPN: Stochastic petri net
SSN: Stochastic symmetric net
ESSN: Extended stochastic symmetric net
SIR: Susceptible-infected-recovered
SIRS: Susceptible-infected-recovered-susceptible
SSA: Stochastic simulation algorithm
SHS: Stochastic hybrid simulation
ODE: Ordinary differential equation
SDE: Stochastic differential equation
PDE: Partial differential equation
CDF: Cumulative distribution function
BDF: Backward differentiation formula
ECDF: Empirical cumulative distribution function
MCS: Monte CarloSampling
aP: acellular Pertussis
wP: whole-cell Pertussis
AIC: Akaike information criterion
ECDC: European centre for disease prevention and control
PRCC: Partial rank correlation coefficient
OP: Optimization problem

## Footnotes

1 Observe that a detailed description of the data sources is reported in subsection *Data information*
