# Supplementary material for: A computational framework for modeling and studying pertussis epidemiology and vaccination
Source: BMC Bioinformatics. 2020 Sep 16;21(Suppl 8):344. doi: 10.1186/s12859-020-03648-6 (PMC7492136; doi:10.1186/s12859-020-03648-6)
Supplement: Supplementary file 1 — Additional file 1 A pdf file providing more information on the usage of the proposed framework, in particular the whole Pertussis analysis is showed. Furthermore, a simpler example of analysis is exploited as a step by step guide of the EPIMOD package. [file 12859_2020_3648_MOESM1_ESM.pdf]

EpiMod: examples of usage

Beccuti Marco, Castagno Paolo, Pernice Simone

Contents

Introduction **1**

How to start **1**

Something to know . . . 2

Cases of study **2**

Simple Example . . . 2

SIR model . . . 2

Model generation . . . 3

Sensitivity analysis . . . 5

Calibration analysis . . . 14

Model Analysis . . . 17

Complex Example . . . 17

Pertussis Model . . . 17

General Transitions . . . 18

Parameters . . . 21

Model Generation . . . 23

Sensitivity analysis . . . 23

Model Calibration . . . 28

Model Analysis . . . 33

References **37**

Introduction

In this document we describe how to use the R library *epimod*. In details, *epimod* implements a new general modeling framework to study epidemiological systems, whose novelties and strengths are:

1. the use of a graphical formalism to simplify the model creation phase;
2. the automatic generation of the deterministic and stochastic process underlying the system under study;

3. the implementation of an R package providing a friendly interface to access the analysis techniques implemented in the framework;
4. a high level of portability and reproducibility granted by the containerization (Veiga Leprevost et al. 2017) of all analysis techniques implemented in the framework;
5. a well-defined schema and related infrastructure to allow users to easily integrate their own analysis workflow in the framework.

The effectiveness of this framework is showed through two case studies, the wellknown and simple SIR model, and much more complex model related to the pertussis epidemiology in Italy.

How to start

Before starting the analysis we have to install (1) GreatSPN GUI, (2) docker, and (3) the R package **devtools** for installing *EPIMOD*. GreatSPN GUI, the graphical editor for drawing Petri Nets formalisms, is available online (link to install GreatSPN), and it can be installed following the steps showed therein. Then, the user must have docker installed on its computer for exploiting the *epimod*'s docker images (for more information on the docker installation see: link to install docker), and to have authorization to execute docker commands reported in the command page of function install docker. To do this the following commands must be executed.

1. Create the docker group.

```
$ sudo groupadd docker
```

2. Add your user to the docker group.

```
$ sudo usermod -aG docker $USER
```

The R package *devtools* has to be installed to run *epimod*:

```
install.packages("devtools")
library(devtools)
install_github("qBioTurin/epimod", dependencies=TRUE)
```

```
library(epimod)
```

Then, the following function must be used to download all the docker images used by *epimod*:

```
downloadContainers()
```

Something to know

All the *epimod* functions print the following information:

- *Docker ID*, that is the CONTAINER ID which is executed by the functon;
- *Docker exit status*, if 0 then the execution completed with success, otherwise an error log file is saved in the working directory.

Cases of study

Simple Example

In this section we provide an example of *epimod* usage throuh a simple case study. In details, the SIR model is the simple case of disease diffusion that we have chosen and in the following subsections show how to use *epimod* to model, analyze and study this case. We refer to (Keeling and Rohani 2011) for all the details. All the files exploited in the analysis are available at github.com/qBioTurin/SIR.

SIR model

The S-I-R models the diffusion of an infection in a population assuming three possible states (or compartments) in which any invidual in the population may move. Specifically, (1) *Susceptible*, individuals unexposed to the disease, (2) *Infected*, individuals currently infected by the disease, and (3) *Recovered*, individuals which were successfully recovered by the infection. To consider the simplest case, we ignore the population demography (i.e., births and deaths of individuals are omitted), thus we consider only two possible events: the infection (passage from *Susceptible* to *Infected*), and the recovery (passage from *Infected* to *Recovered*). We are also assuming to neglect complex pattern of contacts, by considering an homogeneous mixing. From a mathematical point of view, the system behaviors can be investigated by exploiting the deterministic approach (Kurtz 1970) which approximates its dynamics through a system of ordinary differential equations (ODEs):

$$
\begin{aligned}
\frac{dS}{dt} &= -\frac{\beta}{N}SI, \\
\frac{dI}{dt} &= \frac{\beta}{N}SI - \gamma I, \\
\frac{dR}{dt} &= \gamma I,
\end{aligned}
\tag{1}
$$

where:

- S, I, R are the number of susceptible, infected, and recovered individuals, respectively;
- β is the infection rate;
- N is the constant population size;
- γ is the recovery rate, which determines the mean infectious period.

Model generation

The first step is the model construction. Starting with the GreatSPN editor tool it is possible to draw the model using the PN formalism and its generalizations. We recall that the Petri Nets are bipartite graphs in which we have two type of nodes, places and transitions. Graphically, places are represented as circles and those are the variables of our systems. On the other hand, transitions are depicted as rectangles and are the possible events happening in the system. Variables and events (i.e., places and transitions) are connected through arcs, showing what variable(s) is (are) affected by a specific event. For more details we refer to (Marsan et al. 1995).

Therefore, as represented in figure 1, we add one place for each variable of the system (i.e., S, I, and R represent the susceptible, infected, and recovered individuals respectively), and one transition for each possible event (i.e., *Infection* and *Recovery*). Finally, we save the PN model as a file with extension *.PNPRO* .

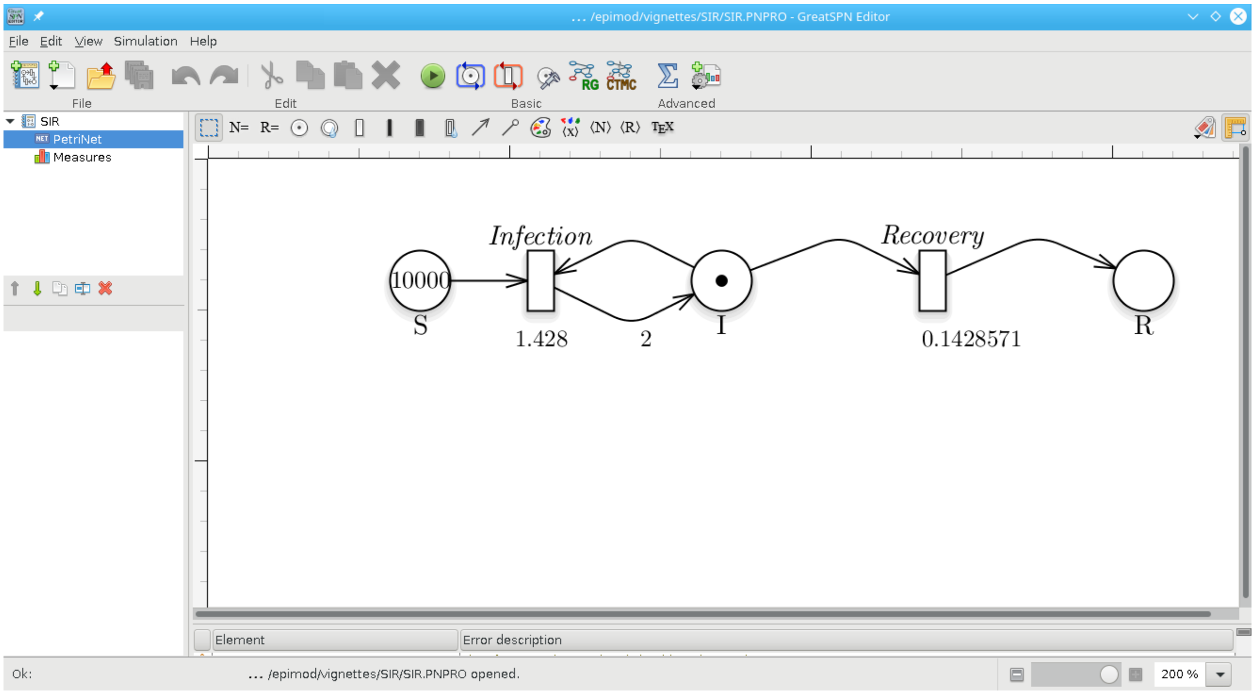

Figure 1: Petri Net representation of the SIR model.

Having constructed the model, the generation of both the stochastic (the Continuous Time Markov Chain) and deterministic (ODEs) processes underlying the model is implemented by the *model_generation()* function. This function takes as input the file generated by the graphical editor, in this case called *SIR.PNPRO*, and automatically derives the processes.

```
model_generation(net_fname = "./Net/SIR.PNPRO")
```

The binary file *SIR.solver* is generated in which the derived processes and the library used for their simulation are packaged.

Notice that *model_generation()* might take as input parameter a C++ file defining the functions characterizing the behavior of general transitions (Pernice et al. 2019), namely *functions_fname*. For instance, if we want to define the transition *Infection* as a general transition then we have to set the transition as *General* and name the corresponding rate name as **FN:NameGeneralFN**, where in this case the *NameGeneralFN* is **InfectionFunction**. As showed in figure 2, where the transition type is set to *General* and the delay (i.e., the rate) to **FN:InfectionFunction**.

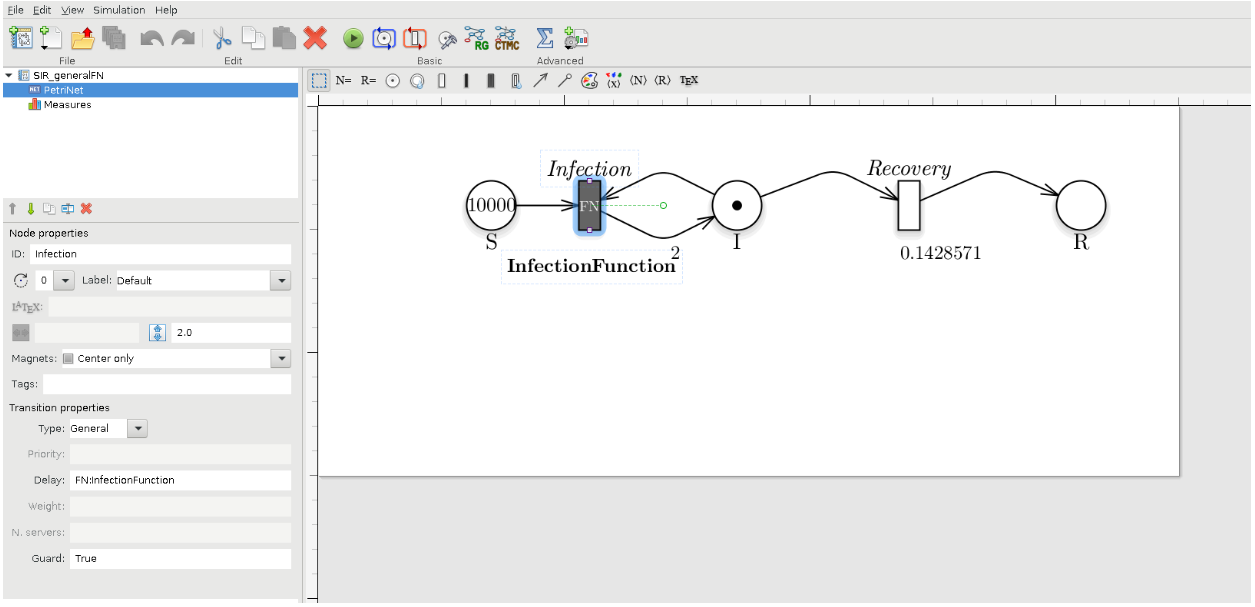

Figure 2: Petri Net representation of the SIR model, modelling the Infection transition as a general transition.

Then, we have to properly define a C++ function implementing the specific behavior of the transition and save it, for instance in a file named *transition.cpp*, which has to be structured as follow:

```
static double Infection_rate = 1.428;

double InfectionFunction(double *Value,
                         map <string,int>& NumTrans,
                         map <string,int>& NumPlaces,
                         const vector<string> & NameTrans,
                         const struct InfTr* Trans,
                         const int T,
                         const double& time)
{

    // Definition of the function exploited to calculate the rate,
    // in this case for semplicity we define it through the Mass Action  law

    double intensity = 1.0;

    for (unsigned int k=0; k<Trans[T].InPlaces.size(); k++)
    {
        intensity *= pow(Value[Trans[T].InPlaces[k].Id],Trans[T].InPlaces[k].Card);
    }

    double rate = Infection_rate * intensity;

    return(rate);
}
```

where the fixed input parameters are:

- **double *Value**: marking of the Petri Net at time *time*;
- **map <string,int>& NumTrans**: association between the transition name and the corresponding index of the *NameTrans* vector;
- **map <string,int>& NumPlaces**: association between the place's name and the corresponding index of the *Value* vector;
- **const vector & NameTrans**: transition names;
- **const struct InfTr* Trans**: array of *InfTr* structures (implemented in GreatSPN) which is indexed using the transition index. The structure *InfTr* has the following fileds: (1) *InPlaces*: the input places to a transition, which is characterized by the input place index position in the *Value* vector (*Trans[T].InPlaces[k].Id*) and the arc (linking the k place with the T transition) molteplicity (*Trans[T].InPlaces[k].Card*). (2) ....
- **const int T**: index of the firing transition;
- **const double& time** : time.

Notice that the function name has to correspond to the rate name associated with the general transition, in this case *InfectionFunction*.

Finally, the process can be derived be the *model_generation()* function as follow.

```
model_generation(net_fname = "./Net/SIR_generalFN.PNPRO",
                 functions_fname = "./Cpp/transition.cpp")
```

Sensitivity analysis

The second step is represented by the sensitivity analysis, in which the deterministic process is solved several times varying the values of the unknown parameters to identify which are the sensitive ones (i.e., those that have a greater effect on the model behavior), by exploiting the Pearson Ranking Correlation Coefficients (PRCCs). This may simplify the calibration step reducing (1) the number of variables to be estimated and (2) the search space associated with each estimated parameter. With this purpose, the function *sensitivity_analysis()* calculates the PRCCs, and, given a reference dataset and a distance measure, it ranks the simulations according to the distance of each solution with respect to the reference one.

In details, the function *sensitivity_analysis()* takes in input

1. **solver_fname**: the *.solver* file generated by the *model_generation* function, that is *SIR.solver*;
2. **n_config**: the total number of samples to be performed, for instance 200;
3. **f_time**: the final solution time, for instance 10 weeks (70 days);
4. **s_time**: the time step defining the frequency at which explicit estimates for the system values are desired, in this case it could be set to 1 day;
5. **parameters_fname**: a textual file in which the parameters to be studied are listed associated with their range of variability. This file is defined by three mandatory columns: (1) a tag representing the parameter type: i for the complete initial marking (or condition), p for a single parameter (either a single rate or initial marking), and g for a rate associated with general transitions (Pernice et al. 2019) (the user must define a file name coherently with the one used in the general transitions file); (2) the name of the transition which is varying (this must correspond to name used in the PN draw in GreatSPN editor), if the complete initial marking is considered (i.e., with tag i) then by default the name *init* is used; (3) the function used for sampling the value of the variable considered, it could be either a R function or an user-defined function (in this case it has to be implemented into the R script passed through the *functions_fname* input parameter). Let us note that the output of this function must have size equal to the length of the varying parameter, that is 1 when tags p or g are used, and the size of the marking (number of places) when i is used. The remaining columns represent the input parameters needed by the functions defined in the third column. An example is given by the file *Functions_list.csv*, where we decided to vary the rates of the *Recovery* and *Infection* transitions

by using the R function which generates values following the uniform probability distribution on the interval from *min* to *max*. We set *n=1* because we must generate one value for each sample.

```
#>   Tag       Name Function Parameter1   Parameter2 Parameter3
#> 1   p   Recovery    runif        n=1      min = 0      max=1
#> 2   p  Infection    runif        n=1  min = 1e-05  max=2e-04
```

Another example might be *Functions_list2.csv*, where we decide to vary the initial marking using the following function *init_generation* defined in the R script *Functions.R* (see *functions_fname* parameter).

```
#>   Tag       Name        Function          Parameter1        Parameter2
#> 1   i       init init_generation min_init = 10000*.8  max_init = 10000
#> 2   p   Recovery           runif                 n=1           min = 0
#> 3   p  Infection           runif                 n=1       min = 1e-05
#>   Parameter3
#> 1
#> 2      max=1
#> 3  max=2e-04
```

6. **functions_fname**: an R file storing the user defined functions to generate instances of the parameters summarized in the *parameters_fname* file. An example is given by *Functions.R*, where the function *init_generation* introduced in *Functions_list2.csv* file is defined in order to sample the initial number of susceptible between *min_init* and *max_init*, and fixing the number of infected and recovered to 1 and 0 respectively.

```
init_generation<-function(min_init , max_init, n)
{
    S=runif(n=1,min=min_init,max=max_init)
# It returns a vector of lenght equal to 3 since the marking is
# defined by the three places: S, I, and R.
    return( c(S, 1,0) )
}
```

7. **target_value_fname**: an R file providing the function to obtain the place or a combination of places from which the PRCCs over the time have to be calculated. In details, the function takes in input a *data.frame*, namely *output*, defined by a number of columns equal to the number of places plus one corresponding to the time, and number of rows equals to number of time steps defined previously. Finally, it must return the column (or a combination of columns) corresponding to the place (or combination of places) for which the PRCCs have to be calculated for each time step. An example is given in *Target.R*, where the PRCCs are calculated with respect to place *I* (infected individuals).

```
Target<-function(output)
{
    I <- output[,"I"]
    return(I)
}
```

8. **reference_data**: a csv file storing the data to be compared with the simulations' result. In *reference_data.csv* we report the SIR evolution starting with 10000 susceptible, one infected and zero recovered, with a recovery and infection rates equals to 0.1428 and 1.428 respectively. Notice that the **reference_data**'s rows must be the variable time serie, and so the colums the corresponding values at a specific time.

```
#>      TimeStep1    TimeStep2    TimeStep3    TimeStep4    TimeStep5    TimeStep6
#> Time         0 1.000000e-01 2.000000e-01 3.000000e-01 4.000000e-01    0.5000000
#> S        10000 9.999848e+03 9.999674e+03 9.999477e+03 9.999253e+03 9998.9984994
#> I            1 1.137136e+00 1.293078e+00 1.470399e+00 1.672030e+00    1.9013056
#> R            0 1.524425e-02 3.257922e-02 5.229125e-02 7.470625e-02    0.1001951
#>         TimeStep7
#> Time    0.6000000
#> S    9998.7088095
#> I       2.1620116
#> R       0.1291789
```

9. **distance_measure_fname**: the R file storing the function to compute the distance (or error) between the model output and the reference dataset itself. The function defining the distance takes in input only the reference data and the simulation's output (i.e. a trajectory); an example is given by *msqd.R* where a distance measure (based on the squared error distance) as function of the infected individuals is defined:

```
msqd<-function(reference, output)
{
    Infect <- output[,"I"]

    diff.Infect <- sum(( Infect - reference )^2 )

    return(diff.Infect)
}
```

Let us observe that: (i) the distance and target functions must have the same name of the corresponding R file,(ii) *sensitivity_analysis* exploits also the parallel processing capabilities, and (iii) if the user is not interested on the ranking calculation then the **distance_measure_fname** and **reference_data** are not necessary and can be omitted.

```
## Simple version where only the transition rates vary.
sensitivity<-sensitivity_analysis(n_config = 200,
                                  parameters_fname = "Input/Functions_list.csv",
                                  solver_fname = "Net/SIR.solver",
                                  reference_data = "Input/reference_data.csv",
                                  distance_measure_fname = "Rfunction/msqd.R" ,
                                  target_value_fname = "Rfunction/Target.R" ,
                                  f_time = 7*10, # weeks
                                  s_time = 1, # days
                                  parallel_processors = 24
                                  )
```

Hence, considering the SIR model we can run the *sensitivity_analysis* varying the *Infection* and *Recovery* transitions rates in order to characterized their effect on the number of infected individuals.

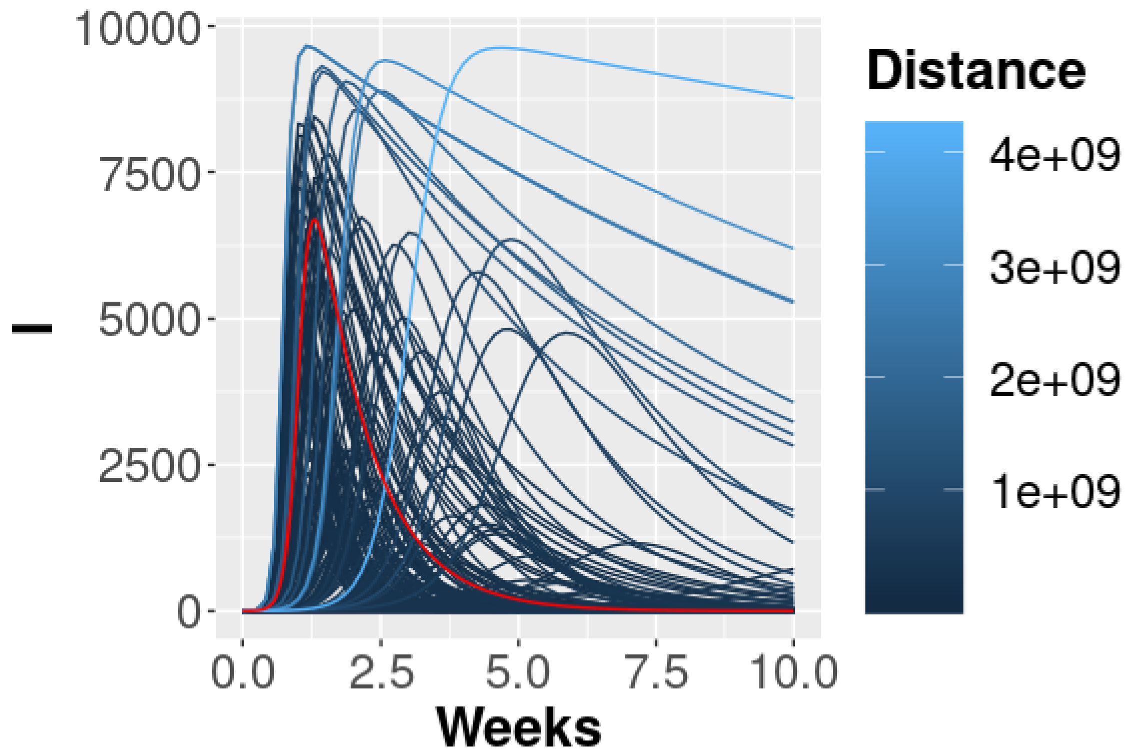

Figure 3: The 200 trajectories considering the I place obtained from different parameters configurations.

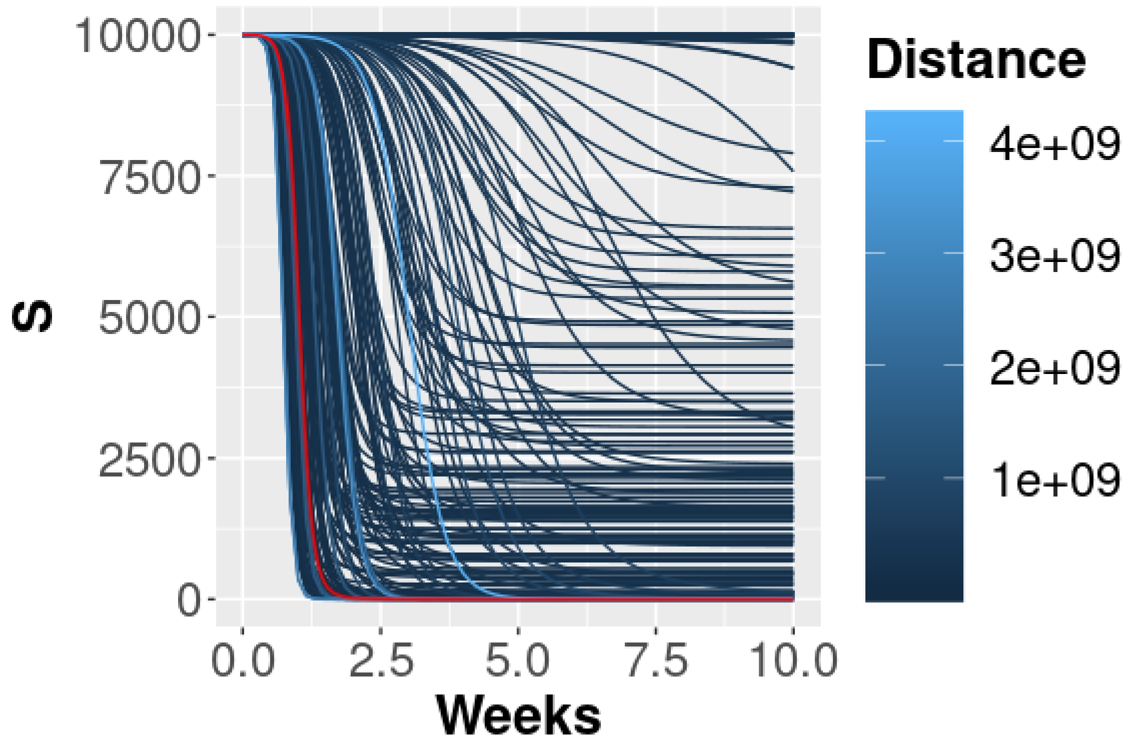

Figure 4: The 200 trajectories considering the S place obtained from different parameters configurations.

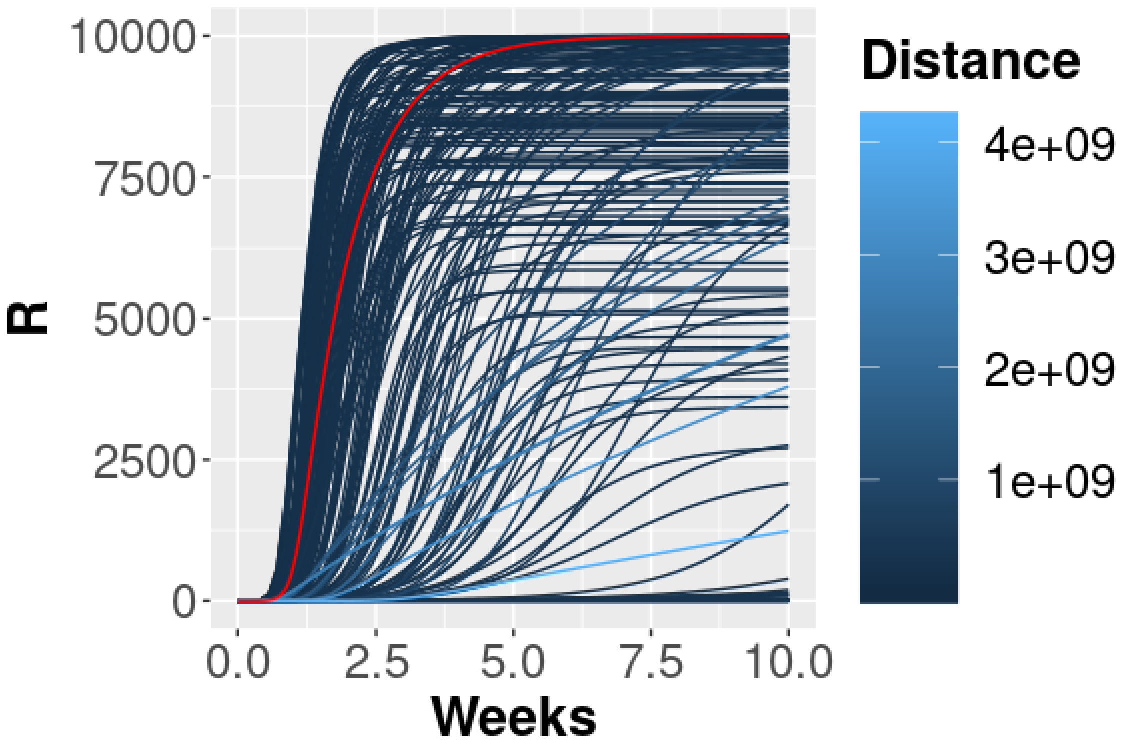

Figure 5: The 200 trajectories considering the R place obtained from different parameters configuration.

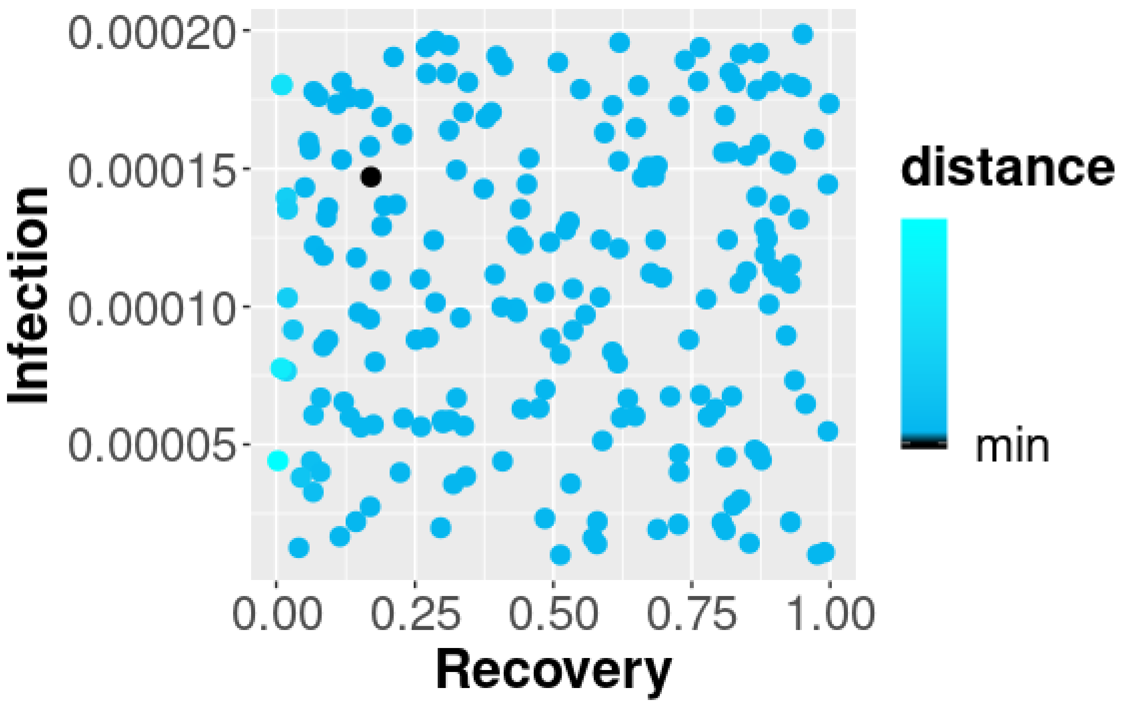

Figure 6: Scatter plot showing the squared error between the reference data and simulated number of infected. The dark blue points represent the parameters configuration with minimum error.

From the figures 3, 5, and 4, it is possible to observe the different trajectories obtained by solving the system of ODEs, represented by eq. 1, with different parameters configurations, sampled by exploiting the function passed through **parameters_fname**. In figure 6 the distance values, obtained using the measure definition described before, are plotted varying the *Recovery* parameter (on the x-axis) and *Infection* parameter (on the y-axis). Each point is colored according to a nonlinear gradient function starting from color dark blue (i.e., lower value) and moving to color light blue (i.e., higher values). From this plot we can observe that lower squared errors are obtained when *Recovery* is around 0.13 and *Infection* around 0.00015, thus we can reduce the search space associated with the two parameters around these two values.

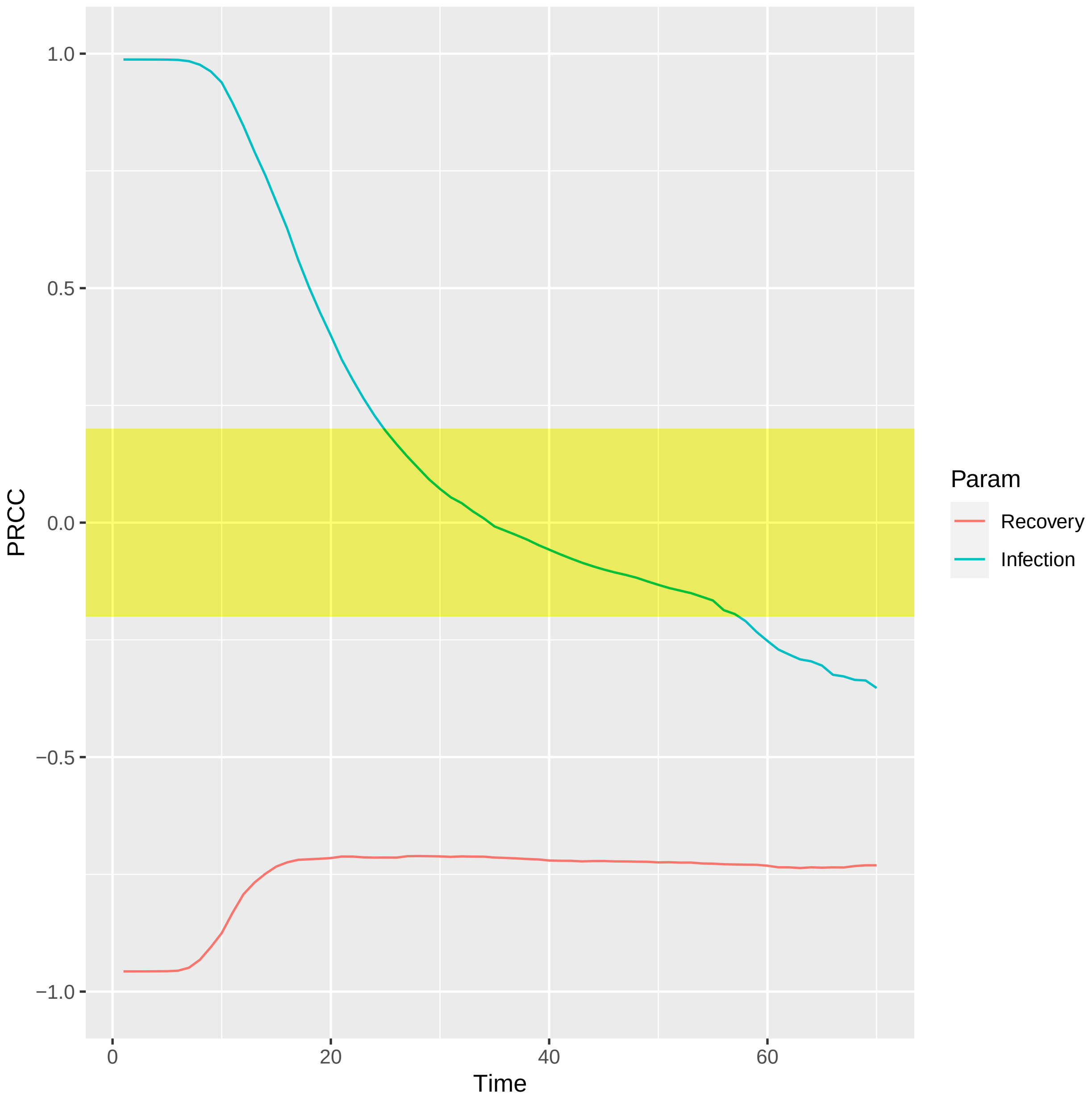

Figure 7: PRCC for the I place over time.

The PRCCs values for these two parameters, depicted in figure 7, with respect the number of infections over the entire simulated period are both meaningful, especially in the first part of the simulation, corresponding to the transient part where the parameters affect mostly the output. Differently, this effect decreases after the fifth week where all the deterministic trajectories obtained with different parameters configurations converge to the same states, see figure 3.

Other possible examples of how to use this function are reported hereafter:

```
## Version where only the PRCC is calculated
sensitivity<-sensitivity_analysis(n_config = 100,
                                  parameters_fname = "Input/Functions_list.csv",
                                  functions_fname = "Rfunction/Functions.R",
```

```
                                    solver_fname = "Net/SIR.solver",
                                    target_value_fname = "Rfunction/Target.R" ,
                                    parallel_processors = 1,
                                    f_time = 7*10, # weeks
                                    s_time = 1 # days
                                    )

## Version where only the ranking is calculated
sensitivity<-sensitivity_analysis(n_config = 100,
                                    parameters_fname = "Input/Functions_list.csv",
                                    functions_fname = "Rfunction/Functions.R",
                                    solver_fname = "Net/SIR.solver",
                                    reference_data = "Input/reference_data.csv",
                                    distance_measure_fname = "Rfunction/msqd.R" ,
                                    parallel_processors = 1,
                                    f_time = 7*10, # weeks
                                    s_time = 1 # days
                                    )

## Complete and more complex version where all the parameters for calculating
## the PRCC and the ranking are considered, and the initial conditions vary too.

sensitivity<-sensitivity_analysis(n_config = 100,
                                    parameters_fname = "Input/Functions_list2.csv",
                                    functions_fname = "Rfunction/Functions.R",
                                    solver_fname = "Net/SIR.solver",
                                    reference_data = "Input/reference_data.csv",
                                    distance_measure_fname = "Rfunction/msqd.R" ,
                                    target_value_fname = "Rfunction/Target.R" ,
                                    parallel_processors = 2,
                                    f_time = 7*10, # weeks
                                    s_time = 1 # days
                                    )
```

Sensitivity analysis with general transitions Let us consider the example of the SIR model where the *Infection* transition is defined as general transition, with the porpoise to varying the *Infection_rate* constant of the corresponding Mass Action law. Generally, in order to define the rate of a transition it is required to provide some inputs and, hence, we need to define an R function (in the **functions_fname** file) which provides all the input parameters necessary to the C++ function.

Therefore, we have to modify the *Functions_list* csv as follow in order to associate with the general transition *Infection* the R function, *InfectionValuesGeneration*, which generates the values exploited by the respective function defined in the C++ file, called *trasition.cpp*.

```
#>   Tag       Name                    Function Parameter1 Parameter2 Parameter3
#> 1   p   Recovery                       runif        n=1    min = 0      max=1
#> 2   g  Infection  InfectionValuesGeneration    min = 0      max=1
```

Successively, we have to define the *InfectionValuesGeneration* in *Functions.R*.

```
InfectionValuesGeneration<-function(min, max)
```

```
{
    rate_value <-  runif(n=1, min = min, max = max)
    return(rate_value)
}
```

Notice that the value (or values) generated are temporarily saved in a file named as the corresponding name in the *Functions_list*, in this case *Infection*. Hence, the file *transition.cpp* has to be modified in order to read and use the value generated from the R function *InfectionValuesGeneration*. An example of implementation is the following, where two functions are defined: (1) *read_constant()* in order to read the generated value, which is associated with the right variable, and (2) *init_data_structures()* in order to read the file only the first time that the function is called.

```
static double Flag = -1;
static double Infection_rate = 1.428;

void read_constant(string fname, double& Infection_rate)
{
    ifstream f (fname);
    string line;
    if(f.is_open())
    {
        int i = 1;
        while (getline(f,line))
        {
            switch(i)
            {
                case 1:
                    Infection_rate = stod(line);
                    //cout << "p" << i << ": " << line << "\t" << p1 << endl;
                    break;
            }
            ++i;
        }
        f.close();
    }
    else
    {
        std::cerr<<"\nUnable to open " << fname <<
                ": file do not exists\": file do not exists\n";
        exit(EXIT_FAILURE);
    }
}

void init_data_structures()
{
    read_constant("./Infection", Infection_rate);
    Flag = 1;

}

double InfectionFunction(double *Value,
                         map <string,int>& NumTrans,
                         map <string,int>& NumPlaces,
```

```
                              const vector<string> & NameTrans,
                              const struct InfTr* Trans,
                              const int T,
                              const double& time)
{

    // Definition of the function exploited to calculate the rate,
    // in this case for semplicity we define it throught the Mass Action  law

    if( Flag == -1)   init_data_structures();

    double intensity = 1.0;

    for (unsigned int k=0; k<Trans[T].InPlaces.size(); k++)
    {
        intensity *= pow(Value[Trans[T].InPlaces[k].Id],Trans[T].InPlaces[k].Card);
    }

    double rate = Infection_rate * intensity;

    return(rate);
}
```

Calibration analysis

The aim of this phase is to optimize the fit of the simulated behavior to the reference data by adjusting the parameters associated with both Recovery and Infection transitions. This step is performed by the function *model_calibration()*, characterized by the solution of an optimization problem in which the distance between the simulated data and the reference data is minimized, according to the definition of distance provided by the user (**distance_fname**)

The function input parameters are very similar to those introduced for the *sensitivity_analysis()*, we have just to modify the **parameters_fname** since we do not need to sample the parameter values. An example is given in *Functions_list_Calibration.csv*, where the first two columns (i.e., type and name) remain unchanged, differently the functions associated with each rate (defined *FunctionCalibration.R*) have to return the value (or a linear transformation) of the vector of the unknown parameters generated from the optimization algorithm, namely x, whose size is equal to number of parameters in **parameters_fname**. Let us note that the output of these functions must return a value for each input parameter.

```
#>   Tag       Name   Function Parameter
#> 1   p   Recovery   recovery       n=1
#> 2   p  Infection  infection       n=1
```

For instance, to calibrate the transition rates associated with *Recovery* and *Infection*, the functions *recovery* and *infection* have to be defined, returning just the corresponding value from the vector x, where *x[1]= "Recovery rate"*, *x[2]= "Infection rate"*, since we do not want to change the vector generated from the optimization algorithm. The order of values in x is given by the order of the parameters in **parameters_fname**.

```
recovery<-function(x)
{
    return(x[1])
}
```

```
infection<-function(x)
{
    return(x[2])
}
```

The remaining parameters are necessary for the optimization process, such as the vector defining the upper/lower bound limits, the initial parameters value, and the control parameters of the optimization (see the R package GenSa (Yang Xiang et al. 2012)).

```
model_calibration(parameters_fname = "Input/Functions_list_Calibration.csv",
                  functions_fname = "Rfunction/FunctionCalibration.R",
                  solver_fname = "Net/SIR.solver",
                  reference_data = "Input/reference_data.csv",
                  distance_measure_fname = "Rfunction/msqd.R" ,
                  f_time = 7*10, # weeks
                  s_time = 1, # days
                  # Vectors to control the optimization
                  ini_v = c(0.15,0.00015),
                  ub_v = c(0.2, 0.0002),
                  lb_v = c(0.1, 0.0001),
                  max.call = 50
                  )
```

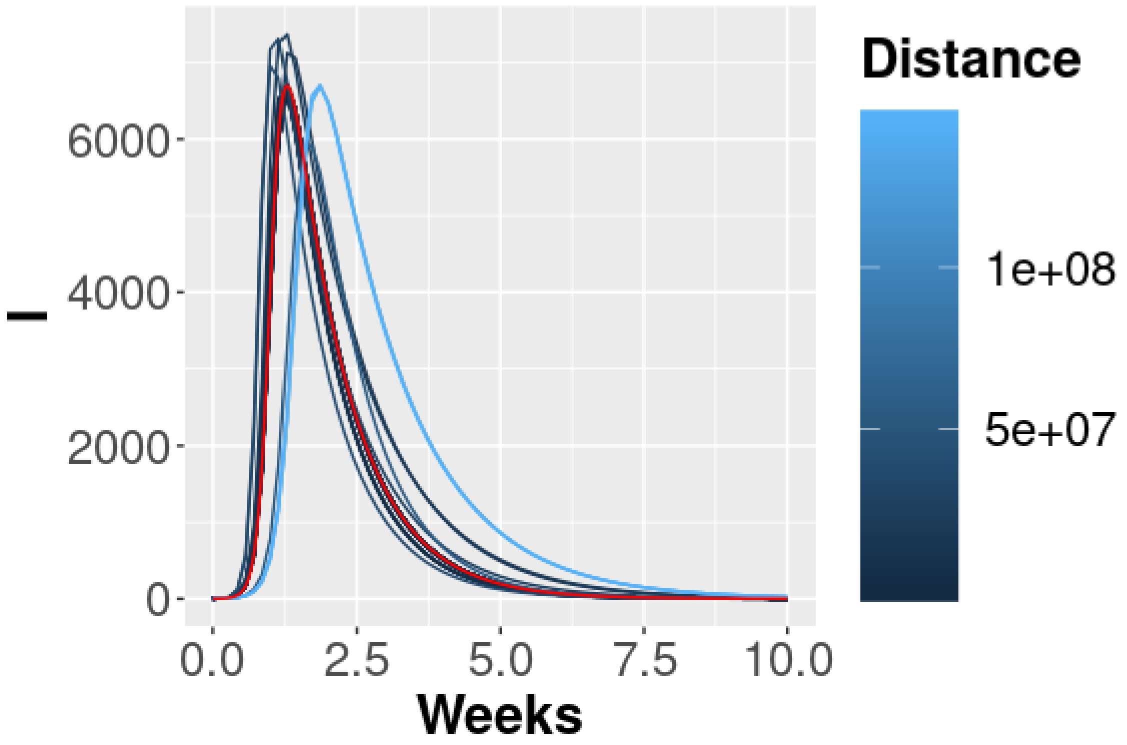

Figure 8: Trajectories considering the I place.

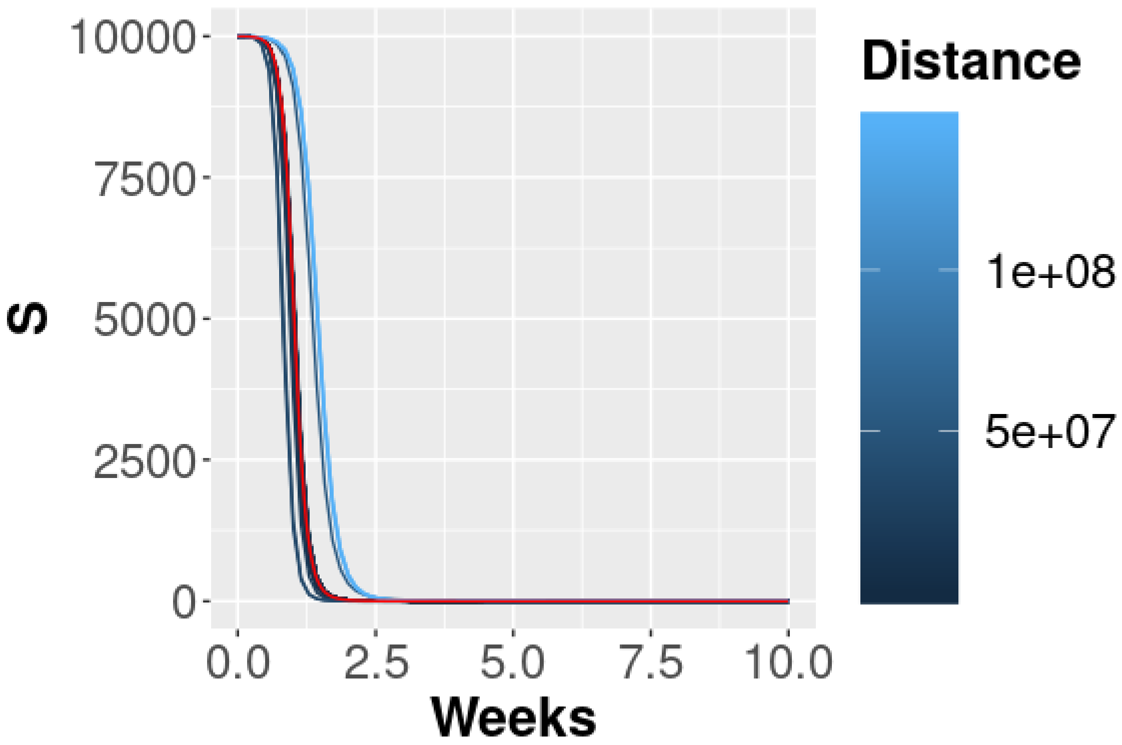

Figure 9: Trajectories considering the S place.

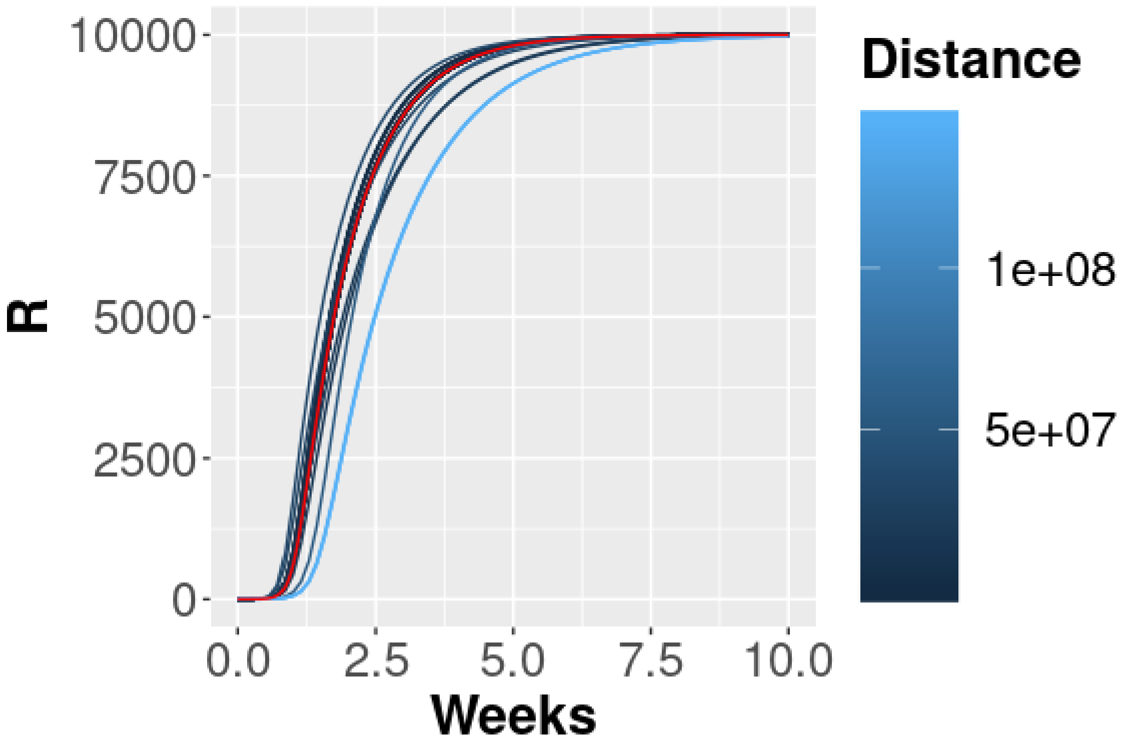

Figure 10: Trajectories considering the R place.

In figures 8,9 and 10 the trajectories with color depending on the squared error w.r.t. reference trend are plotted. In this case, fixing a maximum number of objective function calls, we obtain the following optimal value for the two parameters:

```
#> [1] 0.1428522382 0.0001428038
```

Calibration analysis with general transitions Starting from the changes made in the Sensitivity Analysis phase, we have to add the possibility to save the value passed by the optmization algorithm instead of the value generated by the function defined by the user. By default in the calibration phase, the vector x of the unknown parameters, in this case the *Recovery* and *Infection* rates, is passed to the R functions defined in *Functions.R*. Therefore, we have to modify the *InfectionValuesGeneration* in order to return the value contained in x, i.e. the second one (the order is given by the order of the parameters in **parameters_fname**). Notice that in the Sensitivity Analisys phase, the vector x is not passed in input so we can generalized the *InfectionValuesGeneration* as follow in order to use it in both the analysis phases.

```
InfectionValuesGeneration<-function(min, max, x= NULL)
{
    if(is.null(x))  rate_value <-  runif(n=1, min = min, max = max)
    else rate_value <- x[2]

    return(rate_value)
}
```

Model Analysis

The last step is the model analysis, where the corresponding function *model_analysis()* executes and tests the behavior of the developed model. Furthermore, by changing the input parameters, it is possible to perform a *what-if* analysis or forecasting the evolution of the diffusion process. This function solves the system given a specific parameters configuration which is passed through the function parameter, *parameters_fname*. In this case, instead of writing a function to sample or define the parameter variability, we can pass the specific value obtained from the calibration for generating the corresponding trajectory.

```
#>   Tag       Name Specific value
#> 1   p   Recovery   0.1428522382
#> 2   p  Infection   0.0001428038
```

```
model_analysis(out_fname = "model_analysis",
               solver_fname = "Net/SIR.solver",
               parameters_fname = "Results/Functions_list_ModelAnalysis.csv",
               f_time = 7*10, # weeks
               s_time = 1
               )
```

Complex Example

Pertussis Model

We now describe how the framework functions can be combined to obtain an analysis workflow for the Pertussis model introduced in the main paper: *A computational framework for modeling and studying pertussis epidemiology and vaccination.*

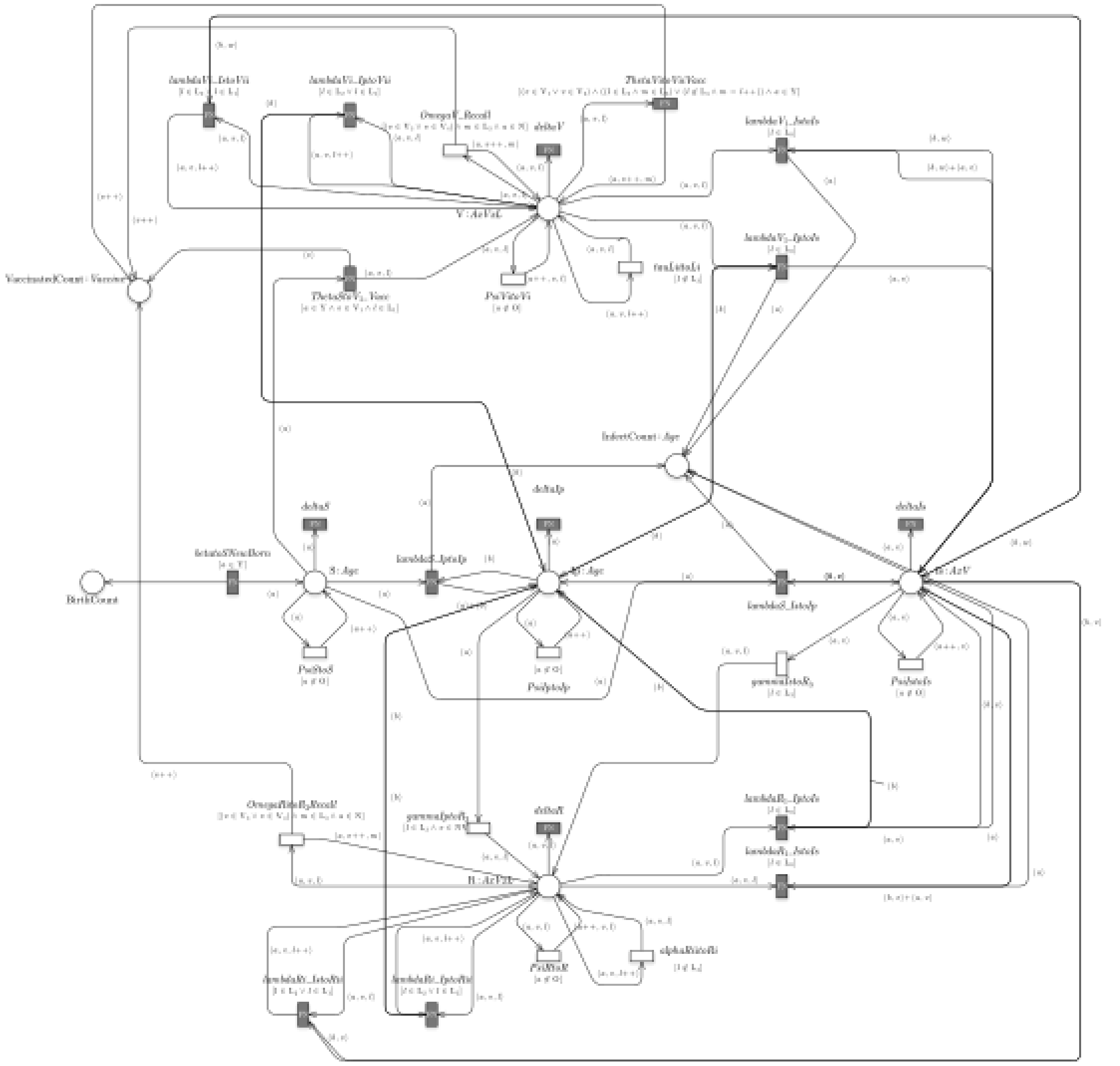

Figure 11: Petri Net representation of the Pertussis model.

We first introduce all the functions, the constants, and the numerical values associated with the general transitions rate (the black boxes in figure 11). Then, we show the proposed framework and how it can be successfully used to study and analyze pertussis infection and the relative vaccination cycle in Italy. All the files exploited in the analysis are available at github.com/qBioTurin/Pertussis.

General Transitions

In this section we describe in details the firing rate function associated with the general transitions in our Pertussis model. Let us recall that:

1. $f_{<t,c>}(\hat{x}(\nu), \nu)$ is the speed of the transition $t \in T_g$ and $\hat{x}(\nu)$ represents the vector of the average number of tokens for all the input places of t. For brevity when the function does not depend on the color instance c then we omit it reporting only the transition t, i.e. $f_t(\hat{x}(\nu), \nu)$.
2. given a transition with a specific color domain, the unfolding procedure generates automatically the transition name combined with all the possible combinations of the color classes associated. For instance, the transition *ContactS_IpToIp* is unfolded into $ContactS\_IpToIp\_a_1\_a_1$, $ContactS\_IpToIp\_a_1\_a_2$, $ContactS\_IpToIp\_a_1\_a_3$, etc.

All the general transitions of the model are now explained in details and all the constants are summarized in Table 1.

Transitions modeling the contacts. All the transitions representing the contact between a person belonging to the age class a_i with one from a_j, which are grouped in the following set:

$$\begin{aligned}T_{contact} = \{&ContactVi\_IpToRii\_a_i\_a_j, ContactVi\_IsToRii\_a_i\_a_j,\\ &ContactV\_IpToIs\_a_i\_a_j, ContactV\_IsToIs\_a_i\_a_j, ContactR\_IpToIs\_a_i\_a_j,\\ &ContactR\_IsToIs\_a_i\_a_j, ContactRi\_IpToRii\_a_i\_a_j, ContactRi\_IsToRii\_a_i\_a_j,\\ &ContactS\_IpToIp\_a_i\_a_j, ContactS\_IsToIp\_a_i\_a_j\}_{i,j=1,2,3}\end{aligned}$$

are defined by the following function:

$$f_t(\hat{x}(\nu), \nu) = prob(t) * \frac{\lambda_{a_i,a_j}}{x^{tot}_{a_j}(\nu)} \hat{x}_i(\nu)\hat{x}_j(\nu) \tag{2}$$

where $t \in T_{contact}$, $x^{tot}_{a_j}(\nu)$ represents the number of people in the a_j class at time ν, λ_{a_i,a_j} the contact rate between the a_i and a_j classes corresponds to the i-row and j-column of the contact matrix 2. $prob(t)$ is a function which returns depending on the transition t if its rate has to be multiplied by a specific probability and it is defined as follows

$$prob(t) = \begin{cases} prob\_infectionS & t \in \{ContactS\_IpToIp\_a_i\_a_j, ContactS\_IsToIp\_a_i\_a_j\} \\ prob\_boost & t \in \{ContactRi\_IpToRii\_a_i\_a_j, ContactRi\_IsToRii\_a_i\_a_j, \\ & \quad ContactVi\_IpToRii\_a_i\_a_j, ContactVi\_IsToRii\_a_i\_a_j\} \\ prob\_infectionR\_l1 & t \in \{ContactV\_IpToIs\_a_i\_a_j, ContactV\_IsToIs\_a_i\_a_j, \\ & \quad ContactR\_IpToIs\_a_i\_a_j, ContactR\_IsToIs\_a_i\_a_j\} \end{cases}$$

Finally,

$$\hat{x}_i(\nu)\hat{x}_j(\nu)$$

is the product of the average numbers of individuals in the the a_i and a_j classes.

Transitions modeling the deaths. Let us define the set of the transitions modeling the death of a person in age class a_i as

$$\begin{aligned}T_{death} = \{&DeathS\_a_i, DeathIp\_a_i, DeathIp\_a_i,\\ &DeathR\_a_i, DeathV\_a_i\}_{i=1,2,3}.\end{aligned}$$

Then we can define the function providing the speed of a transitions $t \in T_{death}$ as

$$f_t(\hat{x}(\nu), \nu) = \delta_{a_i}(\nu)\hat{x}(\nu)$$

where $\delta_{a_i}(\nu)$ is the death rate with respect the age class a_i, and it changes its value depending on the current year:

$$\delta_{a_i}(\nu) = death[i, \nu],$$

where $death[i, \nu]$ is the death rate referred to the year given by ν and the age class a_i considering the rates matrix defined from 1974 to 2016 (columns) for each of three age classes (rows).

Transitions modeling the birth. The birth events are modeled by the transition *Birth*, which is characterized by the following function:

$$f_t(\hat{x}(\nu), \nu) = \mu(\nu)x^{tot}(\nu),$$

where $\mu(\nu)$ is the birth rate per person estimated from 1974 to 2016, obtained from the ISTAT. Since this rate represents the mean number of Italian newborns (in the year ν) per person, it must be multiplied by the total number of Italians (in the year ν), i.e. $x^{tot}(\nu)$.

Transitions modeling the vaccination. The vaccination process starts with the transition *FirstVaccination*, and continues (for the administrations of two further doses) by the *Vaccination* transition. Both of them are characterized by the following function:

$$f_t(\hat{x}(\nu), \nu) = \chi(\nu)\hat{x}(\nu), \tag{3}$$

where $\chi(\nu)$ is administration rate of one vaccine dose, computed as explained in Sec. Vaccination rates. This rate is then multiplied by $\hat{x}(\nu)$, which represents the number of newborns without vaccination, so in the color class NV, (referring to the *FirstVaccination* transition), or the number of newborns already vaccinated one or two times, (referring to the *Vaccination* transition). When the possibility of vaccination failure is also considered, than the function 3 is multiplied to the vaccination failure probability p_v, obtaining the following rates for the *FirstVaccination* and *Vaccination* transitions instead of eq.3:

$$f_t(\hat{x}(\nu), \nu) = \chi(\nu)\hat{x}(\nu)(1 - p_v).$$

To model the vaccination failure further transitions are drawn in the model for simulating the vaccine administrations without an increasing of the resistance level, their rates are defined as follows:

$$f_t(\hat{x}(\nu), \nu) = \chi(\nu)\hat{x}(\nu)(p_v). \tag{4}$$

Parameters

Symbol	*Parameter*	*Value*
γ	Healing rate	21 days
θ^{vacc}	Decay of the resistance derived from vaccination	7 years (21 months per level)
θ^{inf}	Decay of the resistance derived from infection	14 years (42 months per level)
X_0	Initial population distribution	see Sec. *Initial marking*
$\chi(\nu)$	Vaccinations rate (calculated imposing a fixed vaccine coverage)	Minimum of exponential distributions see Sec. *Vaccination rate.*
$\mu(\nu)$	Birth rate depending on the time	Obtained from ISTAT see Sec. *Birth rate.*
$\delta_{a_i}(\nu)$	Death rates depending on time and on the age class	Obtained from ISTAT see Sec. *Death rates.*
$\lambda_{a,b}$	Contact rates between subjects in the age classes a and b	see Table 2
$prob\_boost$	prob. of a natural boost occurrence	To be estimated
$prob\_infetionS$	prob. of infection success of a susceptible	To be estimated
$prob\_infetionR\_l1$	prob. of infection success of a recovered with minimum resistance	To be estimated

Table 1: Parameters of the ESSN model.

Contact rates. We are considering the contact matrix provided by (Mossong 2008), in which the Italian contact rates depending on the age are reported.

	a_1	a_2	a_3
a_1	0.936	1.294	1.39
a_2	0.297405191275416	14.4892996086186	8.62962260197178
a_3	0.118175622978838	3.03663619900113	12.7700507140768

Table 2: Contact rates per age class.

Initial marking. The initial marking is a vector defined by 179 variables, that are all the places of our ESSN associated with all the corresponding color classes combinations (given by the color domain **A**, **V**, and **L**). Since the simulations start from the 1974, from when we are considering no vaccination, all the places with colors different to NV (i.e., no vaccination) are settled to zero. For obvious reasons, all the places

Population clesses	*Value*
Ip_a1	31.98904
Ip_a2	174.8466
Ip_a3	6.415068
Is_a1_nv	31.98904
Is_a2_nv	174.8466
Is_a3_nv	6.415068
S_a1 + R_a1_nv_l4	866703
S_a2 + R_a2_nv_l1+ R_a2_nv_l2 + R_a2_nv_l3 + R_a2_nv_l4	15685693
S_a3 + R_a3_nv_l1+ R_a3_nv_l2 + R_a3_nv_l3 + R_a3_nv_l4	37837299

Table 3: Initial conditions.

representing counters (birth, vaccination, and infection counting) are settled to zero. From (Gonfiantini et al. 2014) and the surveillance data, we were able to estimate the number of infects in each age class in the 1974. In details, during the whole year there were reported 7'400 cases distributed as follow: 15% in N, 80% in Y, and 5% in O. Supposing for simplicity: (1) to split as equals the number of infects between the I_p and I_s places, and (2) to obtain the mean initial number of infects to scale their values with a factor of 21/365, since the healing mean time is 21 days. This is necessary because the time scale of our simulations is *days*.

Finally, the remaining places to estimate their initial markings are given by: the susceptible in each age class (*S_a1, S_a2, S_a3*), and the recovered without vaccination in each age and resistance level class (*R_a1_nv_l4, R_a2_nv_l2, R_a2_nv_l3, R_a2_nv_l4, R_a3_nv_l2, R_a3_nv_l3, R_a3_nv_l4*). For simplicity we consider *R_a1_nv_l1, R_a1_nv_l2, R_a1_nv_l3* equal to zero since the time necessary to decrease the immunity level is greater than 1 year.

Since it is known the size during the 1974 of the Italian populations and how they are distributed among the age classes, removing the infects, we have to estimate through the sensitivity and calibration analysis their distribution among the susceptible and recovered, and the resistance levels as well.

Vaccination rate. The vaccination rate is defined through the vaccination policy and the properties characterizing the Exponential Negative Distribution. So to estimate the vaccination rate χ let us introduce three i.i.d. random variables :

1. death in the first year: $D \sim Exp(\delta_{a_1})$,
2. growth from the first age class to the second one: $G \sim Exp(\lambda_{a_1,a_2})$,
3. vaccination: $V \sim Exp(\chi)$, (equal for each vaccination dose),

and the events (i) $V_i = \{i^{th}\text{vaccination dose is submitted}\}$, $i = 1, 2, 3$, and (ii) $A_1 = \{\text{belonging to the first age class}\}$. Therefore, requiring that the probability to complete the vaccination cycle in absence of disease during first year is equal to p, i.e.

$$\mathbb{P}\{(3 vaccination\ doses\ are\ submitted)|(before\ the\ first\ year)\} = \mathbb{P}\{V_1 \cap V_2 \cap V_3 | A_1\} = p, \tag{5}$$

we are able to deduce that

$$
\begin{aligned}
\mathbb{P}\{V_1 \cap V_2 \cap V_3 | A_1\} = & \\
&= \prod_{i=1}^{3} \mathbb{P}\{V_i | A_1\} \\
&= (\mathbb{P}\{V_1|A_1\})^3 \\
&= (\mathbb{P}\{V = \min(D, G, V)\})^3 \qquad (6)\\
&= (\frac{\chi}{\chi + \delta_{a_1} + \lambda_{a_1,a_2}})^3 = p. \qquad (7)
\end{aligned}
$$

Where we suppose that the random variables $D,\ G, V$ are i.i.d., the three events $V_i,\ i = 1, 2, 3$ as well, and that one vaccination dose is submitted iif the transition modeling the vaccination fires before the transitions modeling the death or the growth (and so the exit form the first age class). Then, the properties characterizing the exponential distributions [1] are exploited to obtain an analytic formula depending on the unknown parameter χ, and the known parameters $\delta_{a_1},\ \lambda_{a_1,a_2},\ p$. Indeed solving the following equation

$$
(\frac{\chi}{\chi + \delta_{a_1}(\nu) + \lambda_{a_1,a_2}})^3 - p(\nu) = 0,
$$

we are able to estimate the vaccinate rate χ depending on the vaccine policy defined a priori. The probabilities p were calculated from the percentage of newborns who completed the vaccination cycle. These percentages were reported every year from 1994 to 2016. For this reason p is time dependent, and they were obtained from (Gonfiantini et al. 2014,@IstatCopVacc).

Birth rate. The birth rate has been computed directly from data provided by ISTAT for the period 1974 $\sim$ 2016. In details, it is defined as the average births per day in the reference period with a dependence on the year.

Death rates. Similarly to the birth rate, the death rates are defined as the average deaths per day from 1974 to 2016, with a dependence on the age class and the year.

Model Generation

The starting point is the derivation from the Pertussis model the corresponding underlying stochastic and deterministic processes by using the function *model_generation*. Then the derived deterministic process is represented by a system of 179 ODEs, while the derived stochastic process is characterized by 1965 possible events. Let us observe that the functions associated with the general transitions (the black boxes in figure 11) are implemented in the file *transitions.cpp*, which is passed as input parameter of the function.

```r
generation <- model_generation(net_fname = "/net/Pertussis.PNPRO",
                               functions_fname = "/cpp/transitions.cpp")
```

Sensitivity analysis

Since the model is characterized by 15 unknown parameters, three of the represent the probabilities of having (i) the *susceptible infection success*, i.e., the infection of a susceptible individual due to a contact

[1] Let $X_1, \ldots, X_n$ be independent random variables, with X_i having an $Exp(\lambda_i)$ distribution, respectively. Then the distribution of $\min(X_1, \ldots, X_n)$ is $Exp(\lambda_1 + \cdots + \lambda_n)$, and the probability that the minimum is X_i is $\frac{\lambda_i}{\lambda_1 + \cdots + \lambda_n}$.

with an infected individual, namely *prob_infectionS*, (ii) the *resistant infection success*, i.e., the infection of a vaccinated or recovered individual with the minimum resistance level due to a contact with an infected individual, namely *prob_infectionR_l1*, and finally (iii) *the natural boosts*, i.e., the restoring of the resistance level to the maximum when a person with resistance level different from the minimum level comes into contact with an infected individual, namely *prob_boost*. The remaining 12 unknown parameters are the initial marking of the susceptible and recovered places. We can apply the function *sensitivity_analysis()* on the deterministic process previously generated and considering the data of the period from 1974 to 1994 as reference targets, in order to identify which parameters are most sensitive w.r.t. the counts of infects. Therefore, the following function input parameters are passed:

1. **solver_fname**: *Pertussis.solver*;
2. **n_config**: the model is run *2^14*;
3. **f_time**: since all the rates were calculated daily and we want to simulate 21 years, then the *f_time* has to be *365*21*;
4. **s_time**: the step time is set to 1 year, *365* days;
5. **parameters_fname**: in *Functions_list.csv* the parameters which have to vary and also the parameters that have to be passed to the general functions stored in *transition.cpp* are reported.

```
#>   Tag          Name        Function
#> 1   g       b_rates          b_rate
#> 2   g       c_rates          c_rate
#> 3   g       d_rates          d_rate
#> 4   g       v_rates          v_rate
#> 5   g probabilities     probability
#> 6   i          init initial_marking
#>                                        Parameter1
#> 1 file='/home/docker/data/input/init_conf.RData'
#> 2 file='/home/docker/data/input/init_conf.RData'
#> 3 file='/home/docker/data/input/init_conf.RData'
#> 4 file='/home/docker/data/input/init_conf.RData'
#> 5 file='/home/docker/data/input/init_conf.RData'
#> 6 file='/home/docker/data/input/init_conf.RData'
```

6. **functions_fname**: in *Functions.R* the functions reported in the third column of *Functions_list.csv* are implemented. For instance, the function associated to the generation of the three probabilities is defined as follows:

```
# if x is null then it means that we have to sample the values by
# exploiting the uniform distribution between 0 and 0.25. Let us note
# that three values corresponding to the three probabilities are
# generated since the uniform intervals are all identical to [0,0.25].
# Differtly when x is not null, then it means the we are using the
# optimization algorithm and the prob. values are already sampled,
# for this reason we take just the first three values of x (the vector
# with size equals to the number of parameter which are varing),
# 0 is given to the probability of vaccination failure that will be used
# in model_analyis.
# The three values obtained are automatically saved in a file called as
# the corresponding name in the Function_list.csv (second column),
# and then read and exploited by the functions in transitions.cpp .

probability <- function(file, x = NULL)
{
```

```
    load(file)
    if( is.null(x) ){
        x <- c(runif(n=1,min=0,max=0.01),
               runif(n=1,min=0,max=0.005),
               runif(n=1,min=0,max=0.01),
               0)
    }
    else{
        x <- c(x[c(1:3)],0)
    }
    return(matrix(x, ncol = 1))
}
```

Let us note that the probabilities values generated are used by the functions modeling all the transitions representing the contact between two individuals (implemented in *transitions.cpp*). For this reason the tag associated with this parameter is g and not p (where p has to be used when a transition rate or a single place is under analysis). Similarly, the function *initial_marking* characterizing the generation of the unknown initial markings of certain places is defined in order to satisfy the following constraints:

$$\begin{aligned} &S\_a1 + R\_a1\_nv\_l4 = 866703 \\ &S\_a2 + R\_a2\_nv\_l1 + R\_a2\_nv\_l2 + R\_a2\_nv\_l3 + R\_a2\_nv\_l4 = 15685693 \\ &S\_a3 + R\_a3\_nv\_l1 + R\_a3\_nv\_l2 + R\_a3\_nv\_l3 + R\_a3\_nv\_l4 = 37837299 \end{aligned} \tag{8}$$

Hence, the values estimated during the sensitivity analysis and model calibration steps are the proportions of the total number in each age class. For instance, the values .4 and .8 might be associated to S_a1 and $R\_a1\_nv\_l4$, meaning that the 0.3333333% (i.e., .4/(.4+.8)) of the total 866703 individuals are in S_a1 and the remaining are in R_a1_nv_l4. For more details regarding the constraints in eqs.8 and the functions associated to the general transitions (in specific how the probabilities are used), see Sec. General transitions, eq. 2. 7. **target_value_fname**: since we are interested to calculate the PRCCs over the time w.r.t. the count of infects per year, the *Select.R* file provides the function to obtain the vector storing the total number of infections per year.

```
Select<-function(output)
{
  ynames <- names(output)
  col_names <- "(InfectCount_a){1}[0-9]{1}"
  col_idxs <- c( which(ynames %in% grep(col_names, ynames, value=T)) )
  # Reshape the vector to a row vector
  ret <- rowSums(output[,col_idxs])
  return(as.data.frame(ret))
}
```

8. **reference_data**: the Pertussis surveillance data from the 1974;

```
#>      Infects
#> 1974    7413
#> 1975   10786
#> 1976   18354
#> 1977    8076
#> 1978   12582
#> 1979   18142
```

9. **distance__measure__fname**: the *(mean square error)* squared error estimator via trajectory matching on the number of cases per year is implemented through the *msqd()* function.

```
msqd<-function(reference, output)
{
    ynames <- names(output)
    # InfectCount is the place that counts how many new infects occurs during the whole
    # period, for this reason we have to do the difference to obtain the number of cases
    # per year. Given this difference the squared error is calculated w.r.t. the reference
    # data.
    col_names <- "(InfectCount_a){1}[0-9]{1}"
    col_idxs <- c( which(ynames %in% grep(col_names, ynames, value=T)) )
    infects <- rowSums(output[,col_idxs])
    infects <- infects[-1]
    diff<-c(infects[1],diff(infects,differences = 1))
    ret <- sum(( diff - reference )^2 )/length(diff)
    return(ret)
}
```

Finally,

```
sensitivity_analysis(n_config = 2^14,
                     parameters_fname = "./Functions_list.csv",
                     functions_fname = "./Functions.R",
                     solver_fname = "./Pertussis.solver",
                     f_time = 365*21,
                     s_time = 365,
                     timeout = "1d",
                     parallel_processors=40,
                     reference_data ="./reference_data.csv",
                     distance_measure_fname="./msqd.R",
                     target_value_fname="./Select.R"
                     )
```

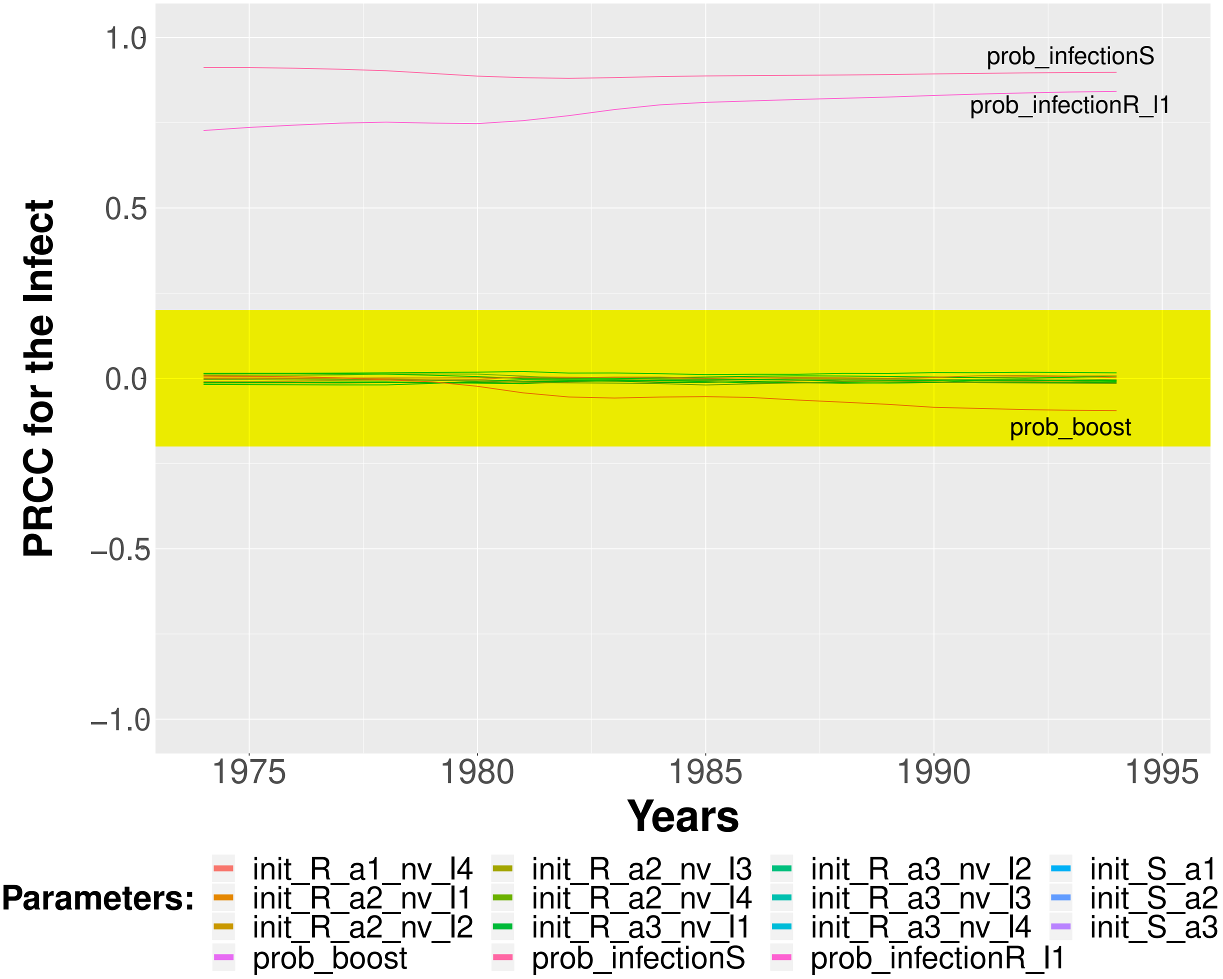

Figure 12: PRCCs values for the selected input parameters with respect the number of infections.

From figure 12 it is straightforward to argue that the *prob_infectionS* is the most important parameter affecting the *infects* behavior, followed by *prob_infectionR_l1*. Differently the *prob_boost* probability and the initial number of susceptible and recovered individuals in each age class are irrelevant with respect to the infection behavior.

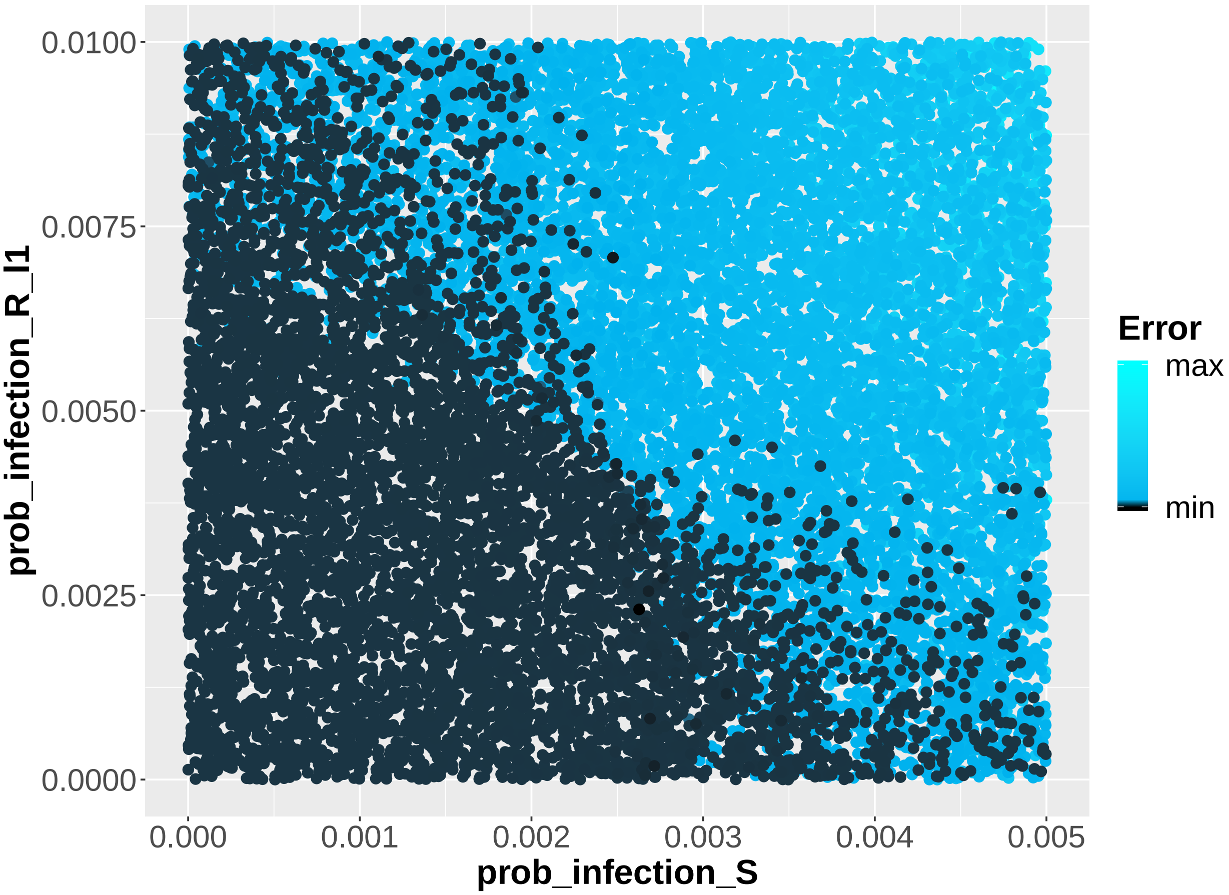

Figure 13: Scatter Plot showing the squared error between the real and simulated infection cases.

In figure 13, the squared error between the real and simulated infection cases from 1974 to 1994 are plotted varying the *prob_infectionS* parameter (on the x-axis) and *prob_infectionR_l1* parameter (on the y-axis). Each point is then colored according to a linear gradient function starting from color dark blue (i.e., lower value) and moving to color light blue (i.e., higher values). From this plot we can observe that higher squared errors are obtained when *prob_infectionS* assumes values greater than 0.0025 and *prob_infectionR_l1* values are greater than 0.005, see light blue points within the quarter identified by values of *prob_infectionS* $\in [0.0025, 0.005]$ and *prob_infectionR_l1* $\in [0.005, 0.01]$. Therefore, according to this we shrunk the search space associated with the two parameter in order to exclude the aforementioned area from the parameters search space in the calibration phase.

Model Calibration

The aim of this phase is to adjust the model unknown parameters to have the best fit of simulated behaviors to the real data, i.e. the reference data. Firstly, the function *model_calibration()* is applied on the generated deterministic process to fit its behavior to the real infection data (from 1974 to 1994) using squared error estimator via trajectory matching implemented in the function *msqd()* passed as an input parameter. Note that the information derived by the sensitivity analysis is exploited to reduce, where it is possible, the number of parameters to be estimated and/or their search space.

```
model_calibration(parameters_fname = "./input/Functions_list.csv",
                  functions_fname = "./Rfunction/Functions.R",
                  solver_fname = "./Net/Pertussis.solver",
                  f_time = 365*21,
                  s_time = 365,
                  reference_data = "./input/reference_data.csv",
                  distance_measure_fname = "./Rfunction/msqd.R",
                  # Vectors to control the optimization
                  ini_v = c(0.0025,0.003,0.0025,
                            1,0,
                            1,0,0,0,0,
                            1,0,0,0,0),
                  ub_v = c(0.0025,0.01,0.0025,
                           1, 1,
                           1, 1, 1, 1, 1,
                           1, 1, 1, 1, 1),
                  lb_v = c(0,0.0025,0,
                           1e-7, 1e-7,
                           1e-7, 1e-7, 1e-7, 1e-7, 1e-7,
                           1e-7, 1e-7, 1e-7, 1e-7, 1e-7),
                  ini_vector_mod = TRUE
)
```

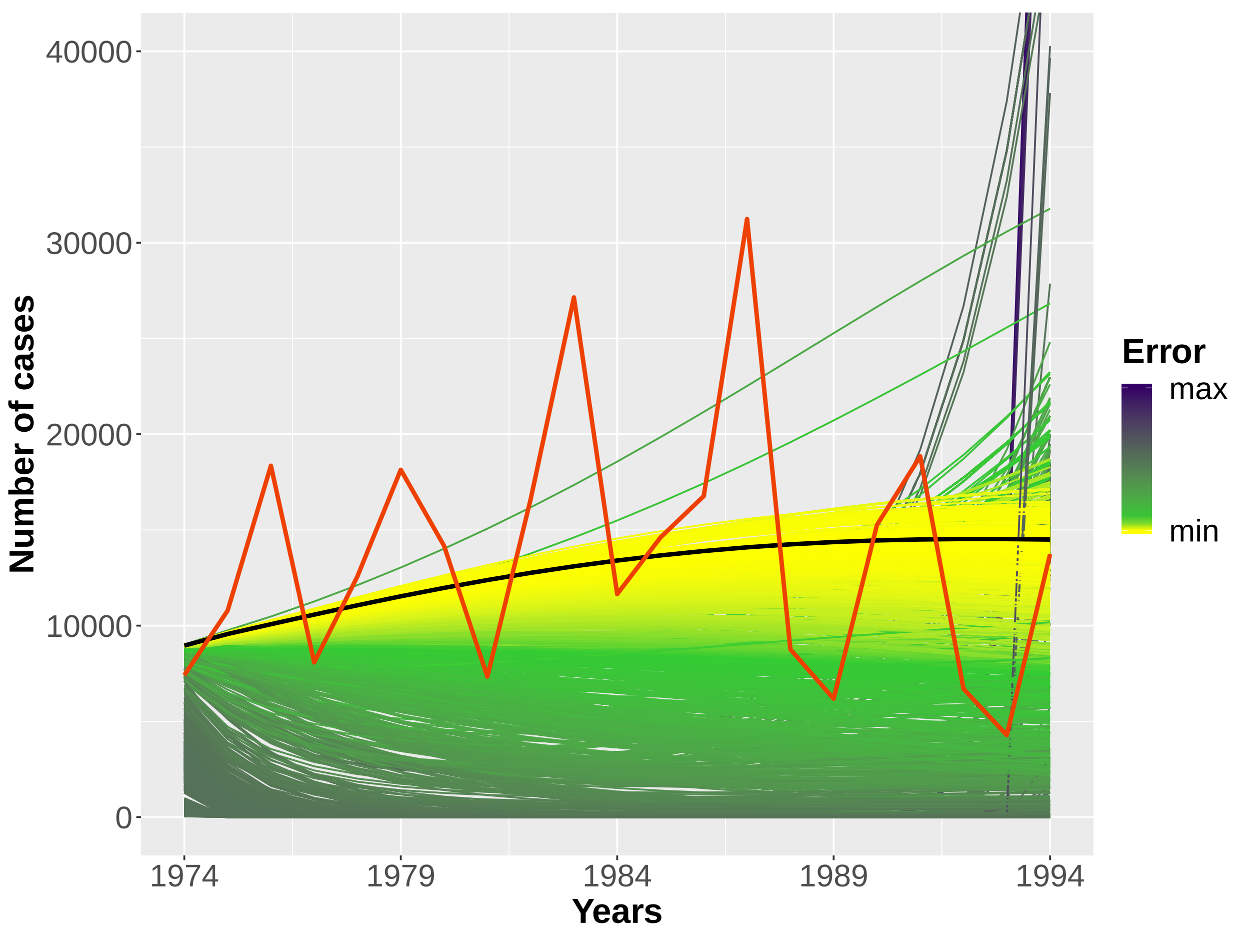

Figure 14: Model Calibration considering the deterministic model. Here a subset of the trajectories obtained from the optimization phase. The color of each trajectory depends on the squared error w.r.t. the Pertussis surveillance trend (red line). The black line is the optimal one.

Then, starting from the parameters configuration obtained from the deterministic model calibration, the function *model_calibration()* is applied on the generated stochastic process to fit its behavior to the real infection data using Akaike Information Criterion (AIC) via trajectory matching, implemented in the R script *aic.R*.

```
# best fitting result from the deterministic model calibration
optim <- c(0.002474758,0.002537443,0.002458887,
           0.931635,7.669116e-06,
           0.9963428,2.133152e-07,1e-07,1e-07,1e-07,
           0.9999986,0.005559236,1e-07,1e-07,1e-07)

model_calibration(parameters_fname = "./input/Functions_list.csv",
                  functions_fname = "./Rfunction/Functions.R",
                  solver_fname = "./Net/Pertussis.solver",
                  f_time = 365*21,
                  s_time = 365,
                  n_run = 2^8,
                  reference_data = "./input/reference_data.csv",
                  distance_measure_fname = "./Rfunction/aic.R",
```

```
                    # Vectors to control the optimization
                    ini_v = optim[1:3],
                    ub_v = c(rep(0.0026,2),0.0025),
                    lb_v = c(rep(0.0025,2),0.0024),
                    ini_vector_mod = TRUE,
                    solver_type = "TAUG",
                    parallel_processors = 16
)
```

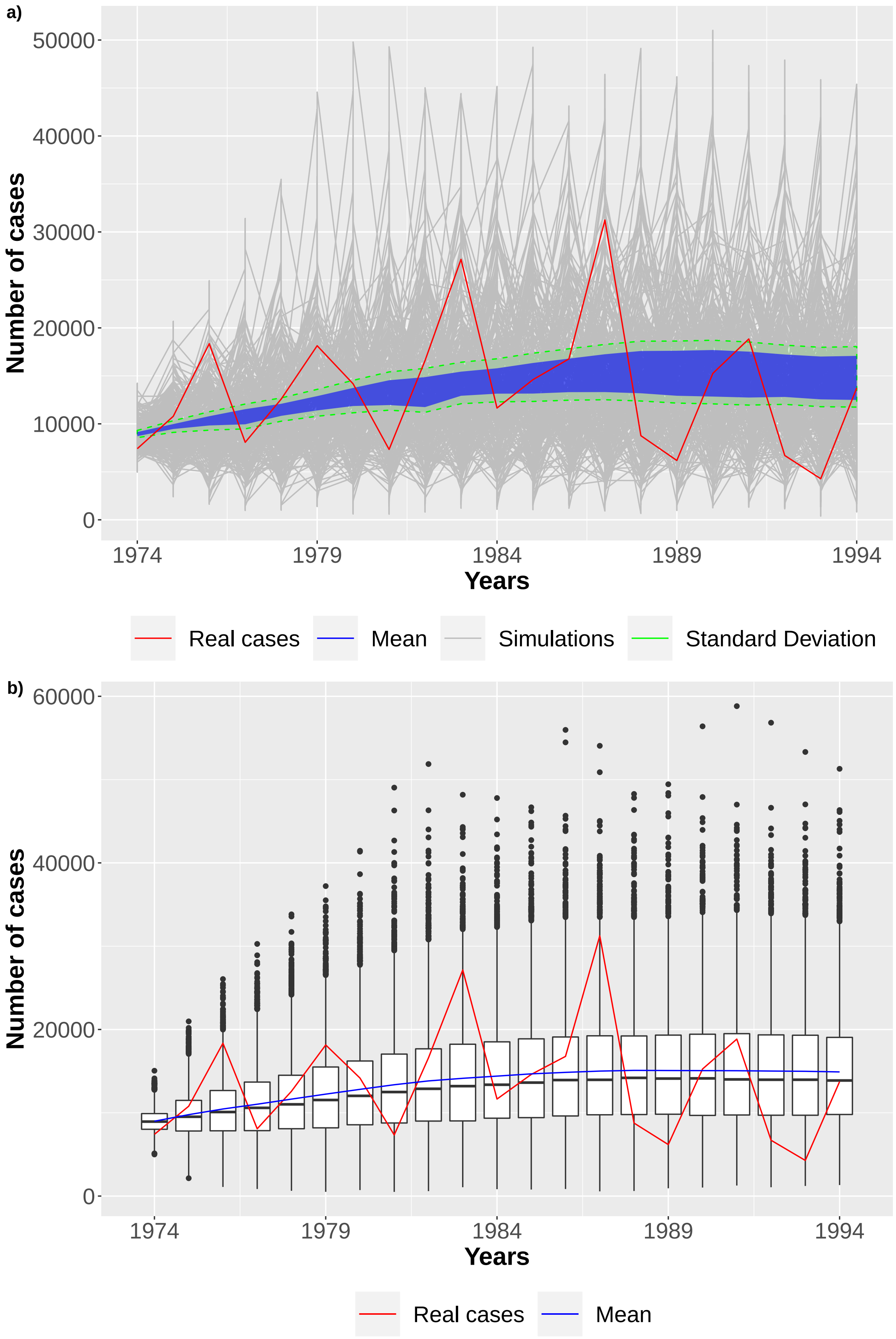

Figure 15: a) 2500 trajectories (grey) over the whole time interval are reported. b) Boxplots considering the best configuration.

Figure 15 shows trajectories (grey lines) for the 15 best parameters configurations discovered. The blue area contains the average trajectories derived for the first ten best parameter configurations, while the two green lines provide the associated confidence interval. We can observe that a good approximation of the surveillance data (red line) from the 1974 to 1994 is obtained.

Model Analysis

In this last phase of our workflow the user can analyse the calibrated model to answer specific questions and to derive new insights. In our case study we show a simple what-if analysis. In particular we investigate the impact of different vaccination failure probabilities with respect to the number of infection cases. The simulated time period is from 1974 to 2016, and the pertussis vaccination program is started in 1995, with an average vaccination coverage starts from 50% and transitions linearly to 95% in 8 years, (Salute 2019),(Gonfiantini et al. 2014).

In details, the *model_analysis()* function is applied by exploiting the optimal parameters configuration derived from the calibration analysis on the stochastic model, namely *optim.stoch*.

```r
# best fitting result from the stochastic model calibration
optim <- c(rnk[1,3:5],
           0.931635,7.669116e-06,
           0.9963428,2.133152e-07,1e-07,1e-07,1e-07,
           0.9999986,0.005559236,1e-07,1e-07,1e-07)

model_analysis(solver_fname = "./Net/Pertussis.solver",
               f_time = 365*43,
               s_time = 365,
               n_config = 1,
               n_run = 2^10,
               parallel_processors = 16,
               solver_type = "TAUG",
               parameters_fname = "./input/Functions_list.csv",
               functions_fname = "./Rfunction/Functions.R",
               ini_v = optim ,
               ini_vector_mod = TRUE)
```

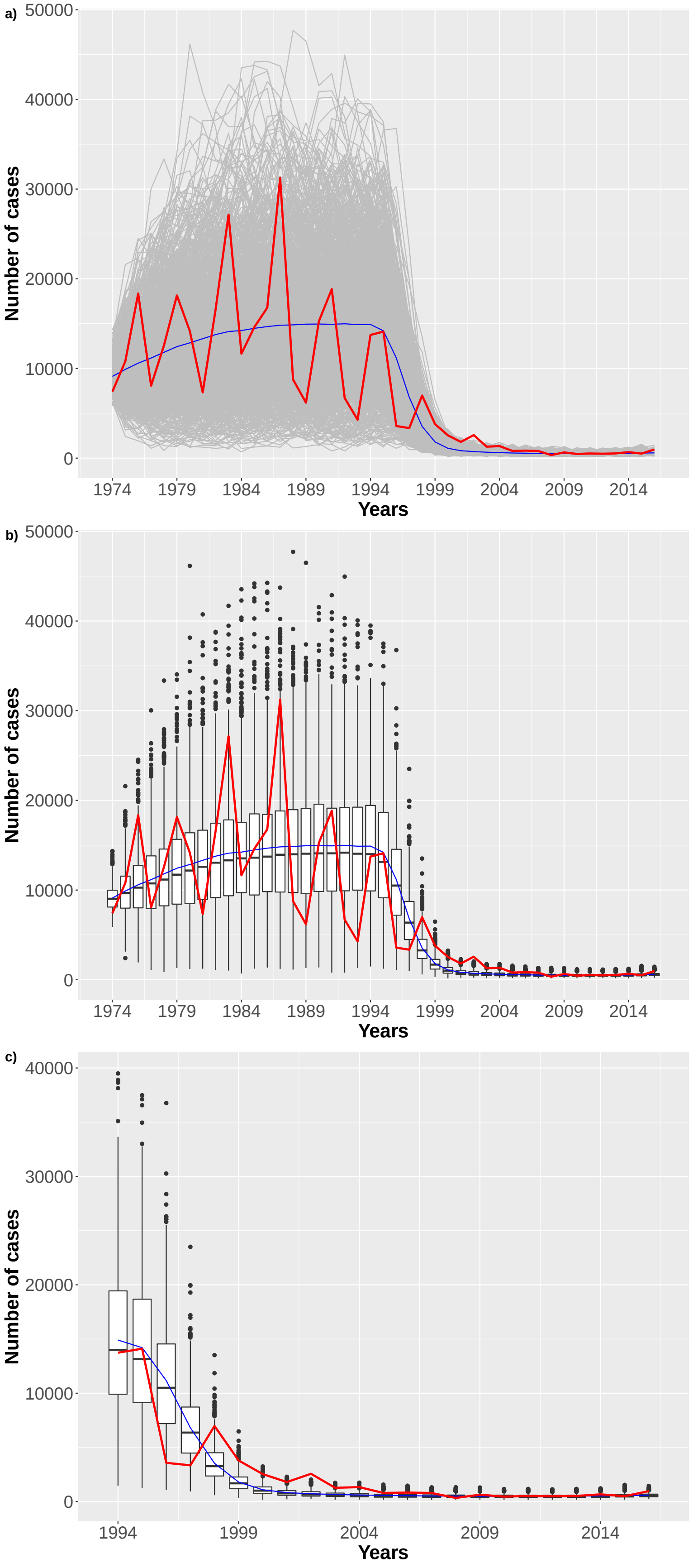

Figure 16: 250 trajectories (grey) considering the stochastic model. The blue dashed line is the mean trend and the red one the Pertussis surveillance. Probability of vaccine failure settled to 0.

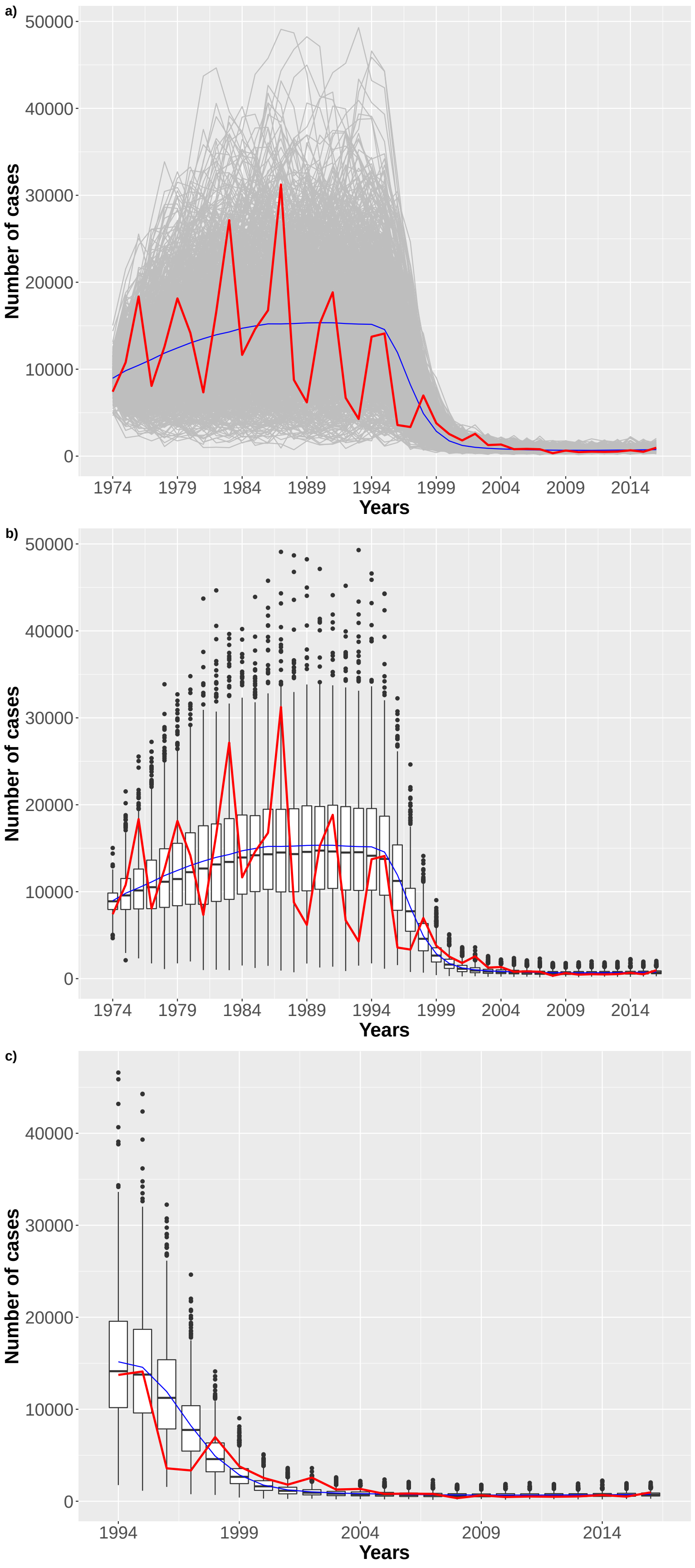

Figure 17: Probability of vaccine failure settled to 0.10.

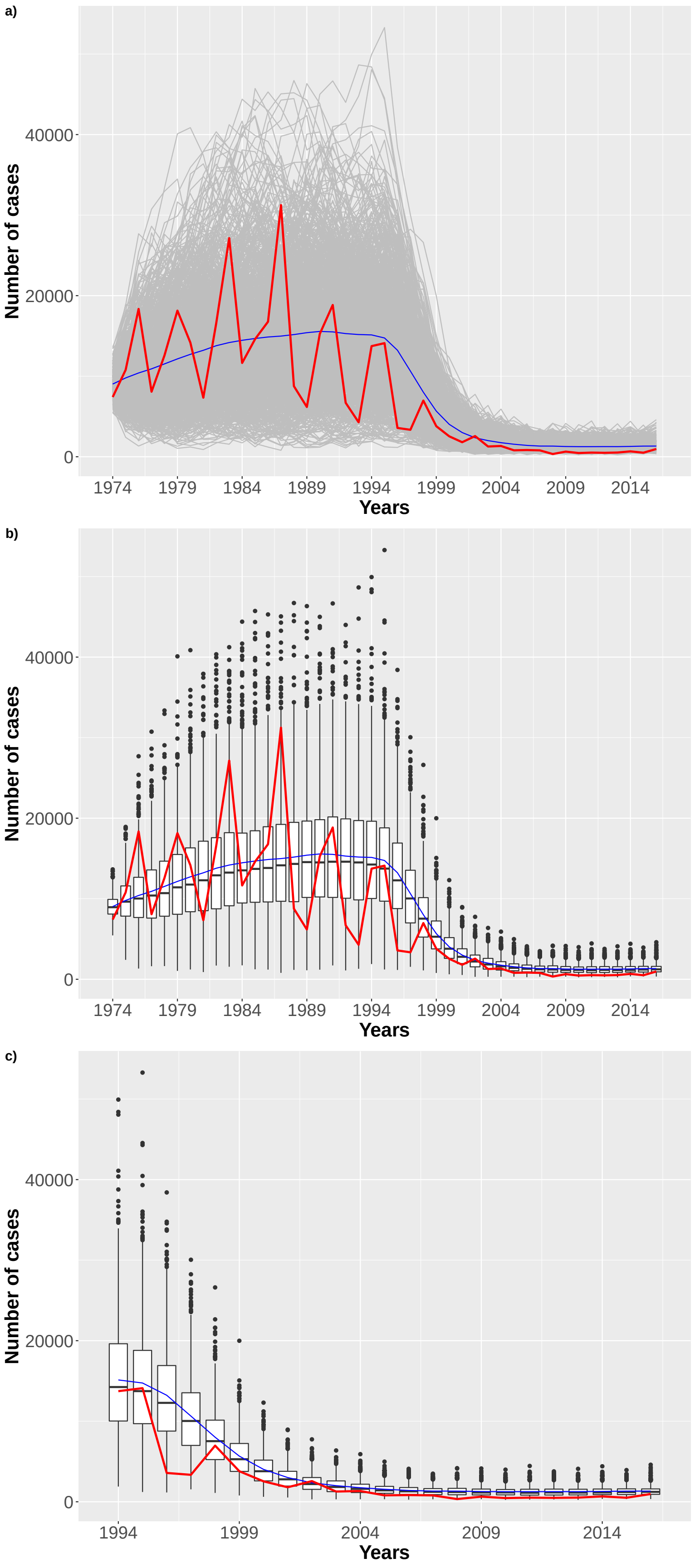

Figure 18: Probability of vaccine failure settled to 0.40.

Fig.16 shows the decreasing of number of infection cases after the starting of the vaccination policy, with dynamics comparable with the reference data. If we add to the model a vaccination failure probability, i.e. we add a fourth probability given by p_v (see Sec.General transitions, eq. 4), then we have to modify the vector returned by the function *probability()* implemented in *Functions.R* as follows:

```
probability <- function(file, x = NULL)
{
    load(file)
    if( is.null(x) ){
        x <- runif(n = length(probabilities), min=0, max=0.25)
        x[length(x)] = 0
    }
    else{
    ##### Here we add the vaccination failure probability: p_v
        x <- c(x[c(1:3)],p_v)
    }
    return(matrix(x, ncol = 1))
}
```

In figures 17 and 18 we show how the number of infection cases is affected by the increasing vaccination failure probabilities from 0.10 to 0.40. We can observe that only probabilities greater than 0.10 have an effect on the number of infection cases.

Figures 16, 17, and 18 show a) 250 trajectories (grey) considering the stochastic model over the whole time interval. The blue dashed line represents the mean trend; the red line represents the Pertussis surveillance trend. In the picture b) the boxplots over the time period are plotted, and in c) the zoom considering the last 21 years is reported.

References

Gonfiantini, M V, E Carloni, F Gesualdo, E Pandolfi, E Agricola, E Rizzuto, S Iannazzo, M L Ciofi Degli Atti, A Villani, and A E Tozzi. 2014. "Epidemiology of Pertussis in Italy: Disease Trends over the Last Century." *Eurosurveillance* 19 (40).

Keeling, Matt J, and Pejman Rohani. 2011. *Modeling Infectious Diseases in Humans and Animals*. Princeton University Press.

Kurtz, T. G. 1970. "Solutions of Ordinary Differential Equations as Limits of Pure Jump Markov Processes." *J. Appl. Probab.* 1 (7): 49–58.

Marsan, M. Ajmone, G. Balbo, G. Conte, S. Donatelli, and G. Franceschinis. 1995. *Modelling with Generalized Stochastic Petri Nets*. New York, NY, USA: J. Wiley.

Mossong, Niel AND Jit, Joël AND Hens. 2008. "Social Contacts and Mixing Patterns Relevant to the Spread of Infectious Diseases." *PLOS Medicine* 5 (3): 1–1. https://doi.org/10.1371/journal.pmed.0050074.

Pernice, S., M. Pennisi, G. Romano, A. Maglione, S. Cutrupi, F. Pappalardo, G. Balbo, M. Beccuti, F. Cordero, and R. A. Calogero. 2019. "A Computational Approach Based on the Colored Petri Net Formalism for Studying Multiple Sclerosis." *BMC Bioinformatics*.

Salute, Ministero della. 2019. "Ministero Della Salute: Coperture Vaccinali."

Veiga Leprevost, Felipe da, Björn A Grüning, Saulo Alves Aflitos, Hannes L Röst, Julian Uszkoreit, Harald Barsnes, Marc Vaudel, et al. 2017. "BioContainers: an open-source and community-driven framework for software standardization." *Bioinformatics* 33 (16): 2580–2.

Yang Xiang, Sylvain Gubian, Brian Suomela, and Julia Hoeng. 2012. "Generalized Simulated Annealing for Efficient Global Optimization: The GenSA Package for R." *The R Journal*. http://journal.r-project.org/.
